# A single-cell, long-read, isoform-resolved case-control study of FTD reveals cell-type-specific and broad splicing dysregulation in human brain

**DOI:** 10.1016/j.celrep.2025.116198

**Published:** 2025-09-05

**Authors:** Natan Belchikov, Wen Hu, Li Fan, Anoushka Joglekar, Yi He, Careen Foord, Julien Jarroux, Justine Hsu, Shaun Pollard, Sadaf Amin, Andrey D. Prjibelski, Shiaoching Gong, Sai Zhang, Roberta Giannelli, Harro Seelaar, Alexandru I. Tomescu, M. Elizabeth Ross, Alissa Nana Li, Lea T. Grinberg, Salvatore Spina, Bruce L. Miller, Johnathan Cooper-Knock, Michael P. Snyder, William W. Seeley, Priyanka Rao-Ruiz, Sabine Spijker, August B. Smit, Claire D. Clelland, Li Gan, Hagen U Tilgner

**Affiliations:** 1 Feil Family Brain and Mind Research Institute, Weill Cornell Medicine, New York, NY, USA; 2 Center for Neurogenetics, Weill Cornell Medicine, New York, NY, USA; 3 Physiology, Biophysics & Systems Biology Program, Weill Cornell Medicine, New York, NY, USA; 4 Helen and Robert Appel Alzheimer’s Disease Research Institute, Weill Cornell Medicine, New York, NY, USA; 5 Department of Computer Science, University of Helsinki, Helsinki, Finland; 6 Department of Epidemiology, University of Florida, Gainesville, FL, USA; 7 Department of Genetics, Stanford University School of Medicine, Stanford, CA, USA; 8 Stanford Center for Genomics and Personalized Medicine, Stanford, CA, USA; 9 Department of Molecular and Cellular Neurobiology, Center for Neurogenomics and Cognitive Research, Amsterdam Neuroscience, VU Amsterdam, Amsterdam, the Netherlands; 10 Department of Neurology and Alzheimer Center Erasmus MC, Erasmus MC University Medical Centre, Rotterdam, the Netherlands; 11 Weill Institute for Neurosciences, Memory and Aging Center, Department of Neurology, University of California, San Francisco, San Francisco, CA, USA; 12 Department of Pathology, University of California, San Francisco, San Francisco, CA, USA; 13 Sheffield Institute for Translational Neuroscience (SITraN), University of Sheffield, Sheffield, UK; 14 These authors contributed equally; 15 Lead contact

## Abstract

Progranulin-deficient frontotemporal dementia (GRN-FTD) is a major cause of familial FTD with TAR DNA-binding protein 43 (TDP-43) pathology, which is linked to exon dysregulation. However, little is known about this dysregulation in glial and neuronal cells. Here, using splice-junction-covering enrichment probes, we introduce single-nuclei long-read RNA sequencing 2 (SnISOr-Seq2), targeting 3,630 high-interest genes without loss of precision, and complete the first single-cell, long-read-resolved case-control study for neurodegeneration. Exons affected by FTD-associated skipping are shorter than those whose inclusion is increased. Up to 30% of cell-(sub)type-specific splicing dysregulation is masked by other cell types or cortical layers. Surprisingly, strong splicing dysregulation events can occur in select but not all cell types. In some cases, a cell type switches in FTD to the splicing pattern of a different cell type. In addition, in separate GRN-FTD samples, the more FTD-prone frontal cortex exhibits more FTD-associated splicing patterns than the occipital cortex. Our methodologies are widely applicable to brain and other diseases.

## In brief

Belchikov et al. conducted a case-control study of splicing in frontotemporal dementia using long-read sequencing. They identified multiple splicing-dysregulation events, including cell-type-specific ones; up to 30% would be masked by other cell types without single-cell resolution. In separate samples, similar differences were seen between more- and less-affected brain regions.

## Graphical Abstract

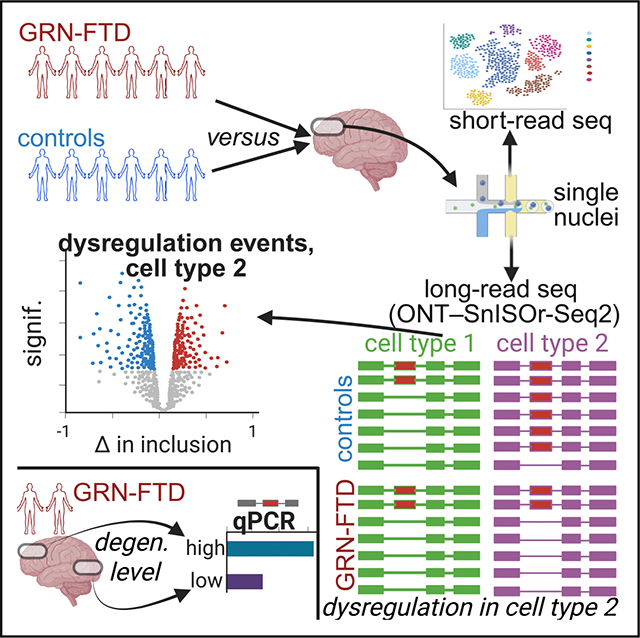

## INTRODUCTION

Frontotemporal dementia (FTD) is a progressive neurodegenerative disorder characterized by the predominant degeneration of the frontal and temporal cortices, with associated deficits in behavior, executive function, and/or language.^1^ Haploinsufficiency of the progranulin (PGRN; *GRN*) gene accounts for 5%–20% of familial FTD and results in nuclear depletion and cytosolic accumulation of the RNA-binding protein TAR DNA-binding protein 43 (TDP-43), which is encoded by the gene *TARDBP*.^2–4^ TDP-43 pathology defines a subgroup of patients with FTD with frontotemporal lobar degeneration (FTLD-TDP) and is also observed in 95% of patients with amyotrophic lateral sclerosis (ALS) and some patients with Alzheimer’s disease (AD). TDP-43 pathology has been connected to the dysregulation of multiple types of splicing patterns, potentially suggesting pathophysiological mechanisms involved in disease initiation and/or spread.^5–16^

While microglia have been implicated in TDP-43 pathology in progranulin-deficient FTLD-TDP (GRN-FTD),^17^ most attention to TDP-43-linked splicing effects has focused on neuronal cells and, more specifically, on neuronal cells with TDP-43 pathology.^12,18^ *TARDBP* is broadly expressed across multiple brain cell types in human and mouse.^19–21^ These observations raise several fundamental questions: (1) does TDP-43 dysregulation affect splicing outcomes similarly or distinctly across cell types and (2) do they occur independently of disease-associated gene expression changes? These questions could be answered in cultured cells or organoids but with the caveat that such models may not recapitulate all aspects of the disease. Human-brain FTD samples and controls acquired postmortem offer a unique window into molecular disease characteristics, both systemically and in cells with TDP-43 pathology,^18^ but retrieving cell-type-specific splicing information out of frozen-brain collections has historically been difficult. Based on the development of long-read RNA sequencing (RNA-seq),^22–24^ as well as single-cell isoform sequencing,^25–27^ we recently developed single-nuclei long-read RNA sequencing (SnISOr-Seq),^28^ which allows analysis of alternative transcription start sites (TSSs), alternative splicing, and alternative polyadenylation (poly(A)) sites in single cells from frozen brain tissue. That approach used exome-targeting probes to select for processed molecules. In the present work, exon-junction-targeting probes were used.

Here, we employ an enhanced SnISOr-Seq protocol for a case-control study in brain tissues from six patients with GRN-FTD and six controls. In all major cell types, we found FTD-associated splicing dysregulation in splice-site and exon usage, a portion of which cannot be detected with bulk RNA-seq. While most splicing alterations tend to occur similarly in multiple cell types, we found strong disease-associated splicing changes specifically in certain cell types or in genes that are preferentially expressed in certain cell types. Moreover, in inhibitory neurons, splicing changes tend to occur in genes that also exhibit dysregulation in gene expression. This association can potentially influence cellular function in a cell-type-specific manner. In summary, this work provides a cell-type-resolved view of splicing alterations in GRN-FTD and a methodology applicable to the investigation of all brain diseases using this single-cell, isoform-resolved technology.

## RESULTS

### Sequencing and quality control

**Figure 1.**
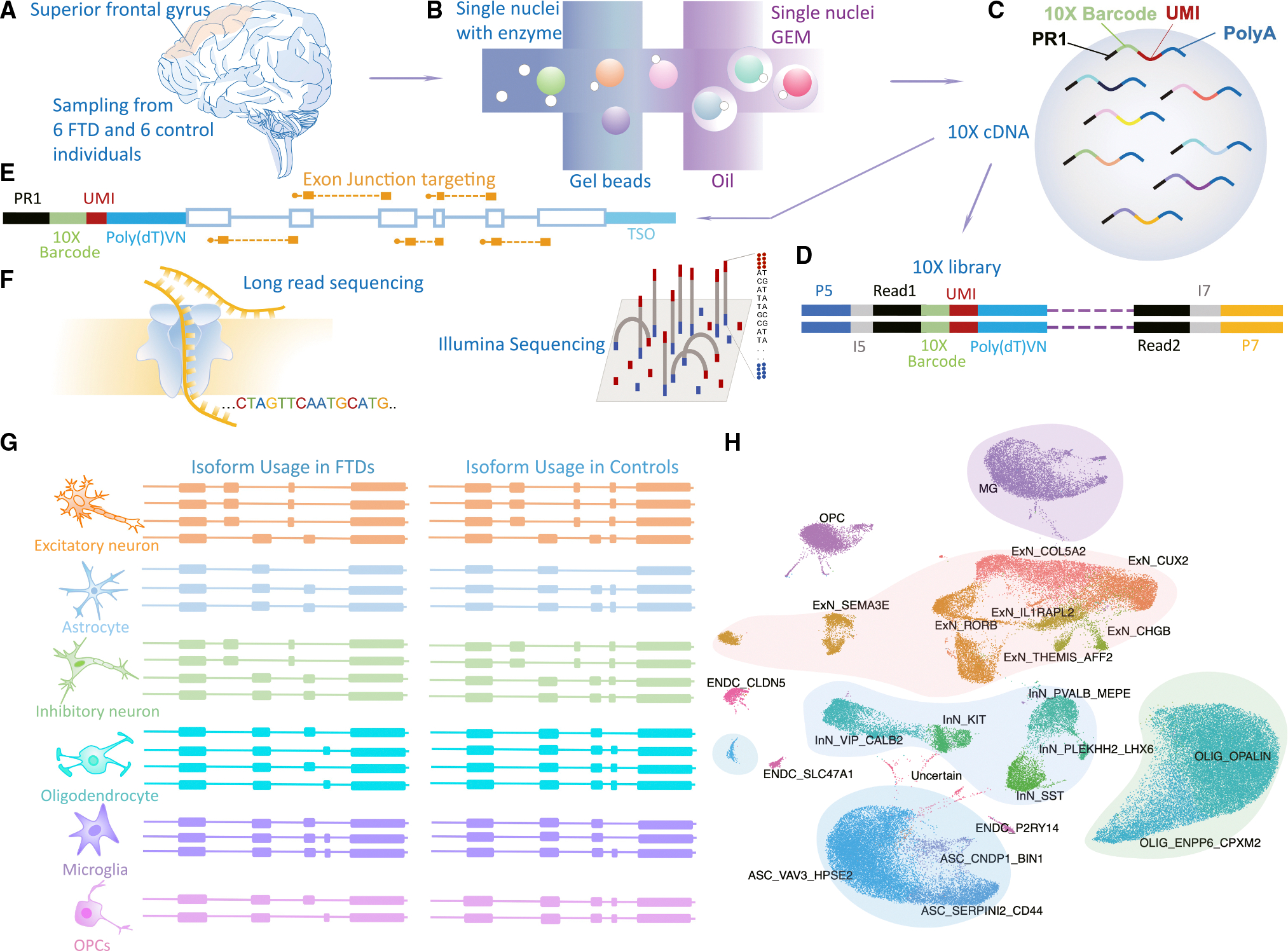
Outline and short-read clustering (A–C) 10× single-nucleus 3′ DNA generation with single-nucleus suspension isolated from six individuals with FTD and six normal control individuals. (D) Construction of 10× single-nucleus 3′ libraries for Illumina sequencing. (E) Enrichment of intronless full-length 10× cDNA with probes targeting exon junctions. (F) Long-read sequencing of cDNA captured from step (E). (G) Identification of differential isoform utilization for each cell type by comparing FTD and control samples. (H) Uniform manifold approximation and projection (UMAP) depiction of clustered cell types for all 12 samples. ASC, astrocytes; ExN, excitatory neurons; InN, inhibitory neurons; OLIG, oligodendrocytes; MG, microglia; OPC, oligodendrocyte precursor cells; ENDC, endothelial cells.

**Figure 2.**
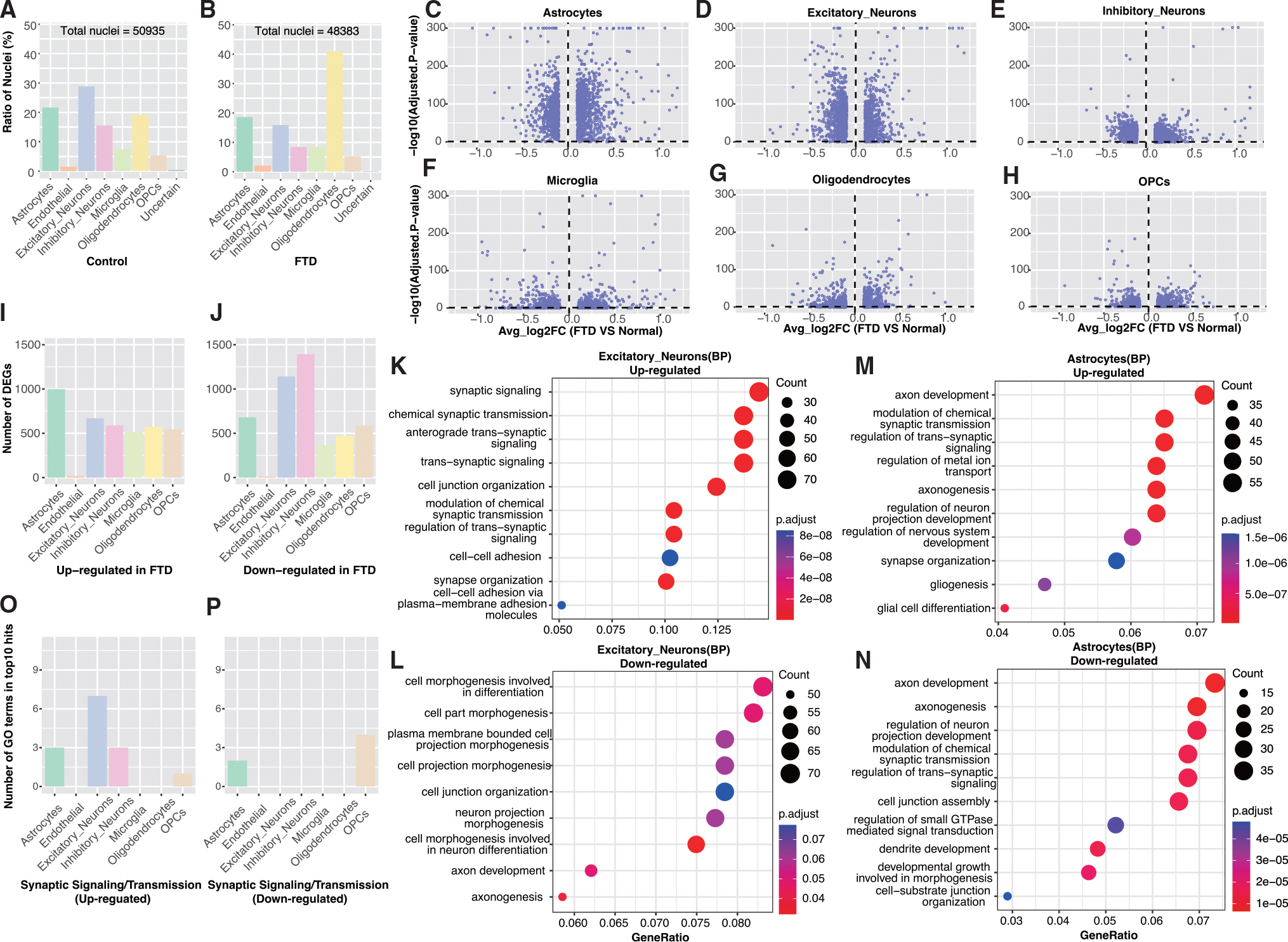
Differential gene expression analysis between FTD samples and controls (A) Proportion of nuclei per cell type in each cell type for controls. (B) Same as (A) but for cases. (C–H) Volcano plots for differential gene expression for six indicated cell types. (I) Number of up-regulated genes, comparing cases to controls in indicated cell types. (J) Same as (I) but for down-regulated genes. (K) GO terms enriched in up-regulated genes (comparing excitatory neurons between cases and controls). (L) Same as (K) but for down-regulated genes. (M) GO terms enriched in up-regulated genes (comparing astrocytes between cases and controls). (N) Same as (M) but for down-regulated genes. (O) Number of synapse-related GO terms in up-regulated genes. (P) Same as (O) but for down-regulated genes.

To investigate the transcriptomic alterations related to GRN-FTD, we obtained superior frontal gyrus samples from six neurologically normal individuals and six patients diagnosed with GRN-FTD (Figure 1A). The subjects ranged in age at death from 56 to 95 years (Table S1). *GRN* mutations influence the function of TDP-43. We first performed single-nucleus sequencing, achieving close to 100,000 high-quality nuclei overall (Figures 1B–1D). An aliquot of unfragmented single-nucleus cDNA was then enriched for barcoded and spliced molecules^28^ (Figure 1E). These enriched cDNAs were then sequenced on the Oxford Nanopore Technologies (ONT) long-read platform and used for differential-isoform analysis between FTD samples and controls (Figures 1F and 1G). In the initial short-read analysis, gene and unique molecular identifier (UMI) numbers per nucleus revealed largely consistent statistics in FTD samples and controls for most broad cell types and subtypes (Figure S1). Using published single-nucleus analysis and clustering methods,^29^ we found all the main cell types expected in a cortical sample, including excitatory and inhibitory neurons, astrocytes, oligodendrocytes, and oligodendrocyte precursor cells (OPCs), as well as microglia and endothelial cells (Figure 1H). We found more oligodendrocytes in FTD samples than in control samples (19.17% in controls and 41.02% in FTD samples; *p* = 0.04, two-sided Wilcoxon rank-sum test) (Figures 2A, 2B, and S2A), mirroring what we have seen in AD.^30^ Although the influence of dissection biases or neuronal loss cannot be excluded, these results may relate to the elevated proliferation of NG2^+^ cells.^31,32^ Considering the two oligodendrocyte subtypes OLIG_OPALIN and OLIG_ENPP6_CPXM2 separately, these accounted for 16.15% and 3.02% in controls and 32.06% and 8.96% in FTD samples, respectively (*p* = 0.04 and 0.06, two-sided Wilcoxon rank-sum test, using proportions for each of the 12 samples) (Figure S2B).

### Gene expression patterns in FTD samples and controls

We first defined up- and down-regulated genes in excitatory neurons, inhibitory neurons, astrocytes, oligodendrocytes, OPCs, microglia, and endothelial cells.^29,33^ Overall, changes were modest, with most significant genes exhibiting less than a 2-fold change in expression for all cell types (Figures 2C–2H). Importantly, the number of differentially expressed genes did not strongly increase with the number of cells per cell type considered. This observation is exemplified by inhibitory neurons, which showed the highest number of down-regulated genes despite having lower cell numbers than excitatory neurons. For excitatory and inhibitory neurons as well as OPCs, down-regulated genes outnumbered up-regulated genes, but the opposite was true for oligodendrocytes and astrocytes (Figures 2I and 2J). Gene Ontology (GO) analysis using ClusterProfiler 4.0^34^ revealed a strong enrichment in synapse-related terms in genes up-regulated in FTD excitatory neurons (Figure 2K), whereas genes down-regulated in FTD excitatory neurons were associated with morphogenesis and differentiation (Figure 2L). GO analysis was performed separately in each cell type using a background set consisting of genes consistently expressed in that cell type (Figure S3). Importantly, synapse- and axon-related GO terms were also enriched in dysregulated genes in astrocytes (Figures 2M and 2N). Thus, multiple cell types, including excitatory neurons, showed synapse- and axon-related GO terms among the top 10 GO terms (Figures 2O and 2P). The synapsin I (*SYN1*) and synaptotagmin 1 (*SYT1*) genes exemplify this synapse-related dysregulation of genes in FTD. Both genes yield high expression counts in control excitatory cells but almost 1.4-fold higher counts in FTD samples (Figure S4).

### Detecting splicing dysregulation in multiple neural cell types

**Figure 3.**
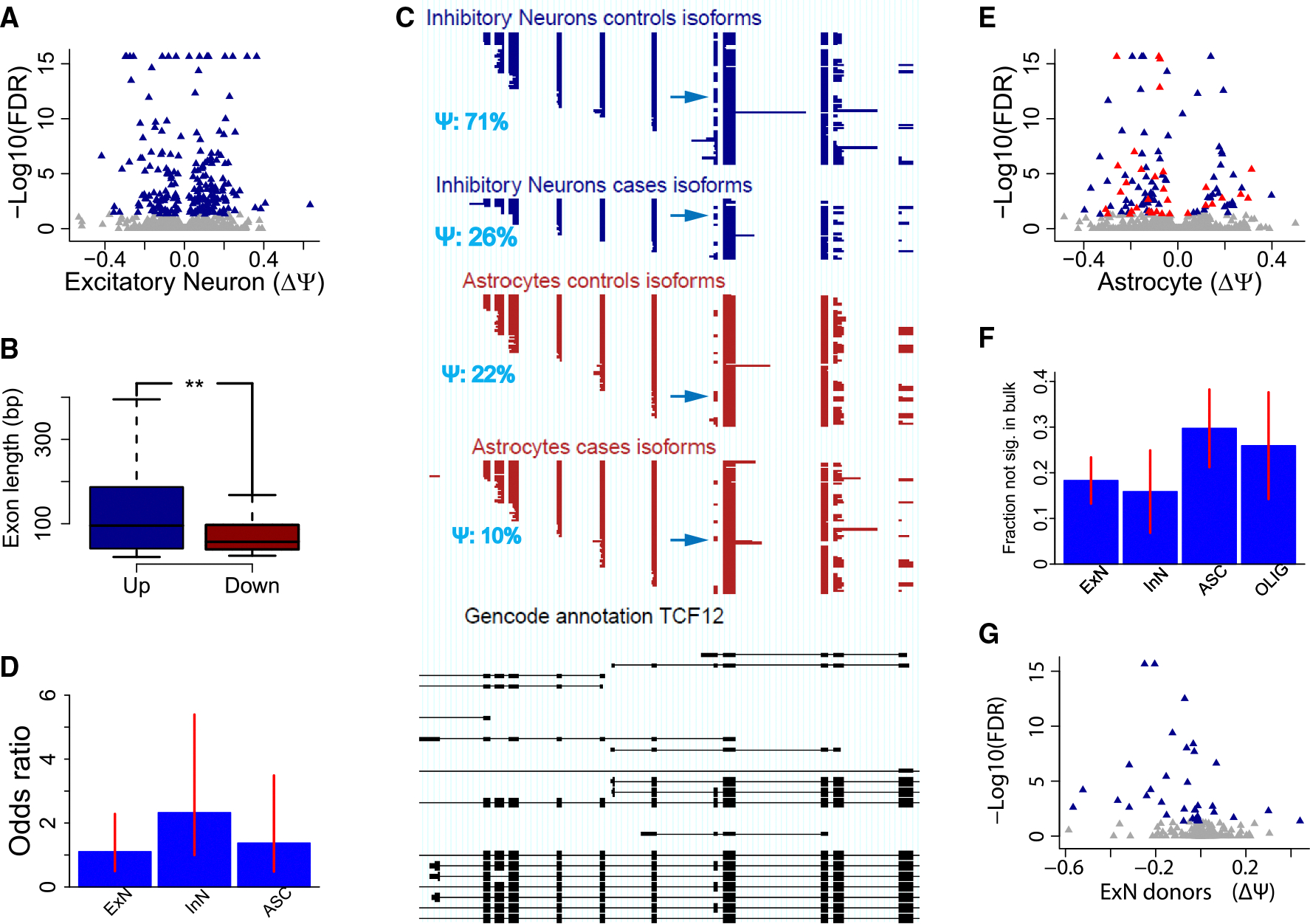
Differential splicing analysis between FTD samples and controls (A) Volcano plot of excitatory-neuron exon inclusion of cases vs. controls. *x* axis: ΔΨ for each exon; *y* axis: −log_10_ (adjusted *p* value). (B) Exon-length distributions for exons with significantly higher inclusion (ΔΨ ≥ 20%) in cases compared to exons with significantly lower inclusion in cases (ΔΨ ≤ −20%). (C) Inhibitory neuron (top tracks in blue) and astrocyte data (bottom tracks in red) for *TCF12*. Each line corresponds to one molecule. Reads from all case samples are grouped together; reads from all control samples are grouped together. Only informative reads for the highlighted exon are shown. Bottom (black) track: GENCODE annotation (v.34) for *TCF12*. (D) Odds ratios for the overlap between differentially expressed genes and genes with significant changes in alternative exon usage between FTD samples and controls. Error bars indicate 95% confidence intervals. (E) Volcano plot of astrocyte exon inclusion in cases vs. controls. Blue points represent exons that were also significant in bulk data; red points represent exons that were only significant in astrocytes. (F) Fraction of the significant exons in each cell type that were not visible in pseudobulk data. Error bars indicate 95% confidence intervals. (G) Volcano plot of alternative donor splice site usage in excitatory neurons for cases vs. controls.

Based on our recent SnISOr-Seq method,^28^ here we devised SnISOr-Seq2, which is based on an enrichment array for 3,630 genes, with probes spanning splice junctions, including genes with known TDP-43 binding,^35^ synaptic genes,^36^ genes with known AD-^37^ and ALS-associated splicing dysregulation,^16^ and genes with highly variable exons,^28^ and, as controls, autism spectrum disorder-associated^38–40^ and schizophrenia-associated splicing dysregulation.^41^ All possible exon-exon junctions based on all annotated transcripts for each of the target genes were targeted by probes, unless technically infeasible. SnISOr-Seq2 outperforms SnISOr-Seq in terms of spliced-molecule recovery among barcoded molecules (Figure S5A) and has no substantial loss in on-target rate, despite using a 5-fold more refined gene set (Figure S5B). Quantifying exon-inclusion differences in cell types with this novel approach correlated highly with our published^28^ approach (Figure S6A). Likewise, differentially expressed genes between cases and controls showed high correspondence between short- and long-read data, further supporting the validity of the long-read approach (Figure S6B). For each cell type, we first identified internal exons that are alternatively spliced in our dataset. We then pooled all FTD reads into one FTD-representing group and all control reads into one control-representing group and counted the number of reads in each condition that supported the inclusion or the exclusion of the internal exons, leading to a 2 × 2 contingency table. Using Fisher’s exact test and the Benjamini-Yekutieli correction^42^ and calculating “percent spliced in” (Ψ) values for each exon, we identified an initial set of alternative exons with |ΔΨ| ≥ 20% and a false discovery rate (FDR) of 0.05. ΔΨ was defined as the control Ψ subtracted from the case Ψ, meaning that a negative ΔΨ value indicates more skipping of an exon in FTD compared to controls, while a positive value indicates more inclusion than in controls. To mitigate the effects of individual-sample variability, we required that at least two-thirds of samples per group (FTD and control) contribute at least one informative read. In addition, if the pooled control Ψ was greater than the pooled FTD Ψ, at least two-thirds of the individual control samples’ Ψ values had to be greater than two-thirds of the individual FTD samples’ Ψ values, or vice versa if the order of the pooled values was reversed. Exons that fulfilled all of these requirements were considered to be significantly dysregulated in FTD. This resulted in 47 dysregulated exons in excitatory neurons, 29 in inhibitory neurons, 32 in astrocytes, 15 in oligodendrocytes, and 3 in microglia. The maximal dysregulation for excitatory neurons corresponded to ΔΨ values of 63% and −42%. An additional 177 excitatory-neuron exons showed small ΔΨ values (between −20% and 20%) (Figure 3A).

To account for the larger average age of our control samples compared to our FTD samples (78.8 vs. 67.5 years), we checked whether ΔΨ values for the dysregulated exons calculated with just the four samples from each group that allowed for a better match in mean age (71.5 years for the FTD group and 71.8 for the control group) were at least 50% of the original ΔΨ values. All dysregulated exons from inhibitory neurons, astrocytes, and microglia satisfied this requirement, as did 98% of exons from excitatory neurons and 93% of those from oligodendrocytes (Table S2).

Exons with increased inclusion in the FTD samples were significantly longer, with a median size of 95 bp, than those having lower inclusion rates in FTD, which had a median exon size of 57 bp (*p* = 0.004, two-sided Wilcoxon rank-sum test) (Figure 3B). One example of dysregulation in several cell types that demonstrates this tendency of shorter exons to be skipped in FTD occurs in a 72-base exon in the transcription factor 12 (*TCF12*) gene. The exon has a 71% Ψ value in both excitatory-neuron and inhibitory-neuron controls but decreases to 29% and 26%, respectively, in FTD samples. Notably, the Ψ is 22% in astrocyte control samples, potentially suggesting that neurons in FTD were switching to a more astrocyte-like splicing pattern (Figures 3C and S7). This behavior was validated by semi-quantitative reverse-transcription PCR (RT-sqPCR), where NeuN^+^ cells showed a marked FTD-specific reduction in inclusion of this alternative exon, while NeuN^−^ cells showed low inclusion in both FTD and control samples (Figure S8C).

Interestingly, exons with significantly different inclusion levels between FTD samples and controls in inhibitory neurons showed a tendency to occur in genes with significantly altered expression (inhibitory-neuron odds ratio of 2.33, *p* = 0.03; two-sided Fisher’s exact test) (Figure 3D). A clear association between the direction of change in expression and splicing was not observed.

### Masking of cell-type-specific dysregulation in pseudobulk

We then asked whether exons altered in the five major cell types (excitatory neurons, inhibitory neurons, astrocytes, oligodendrocytes, and microglia) would have been found in a pseudobulk comparison of cases and controls (i.e., a comparison of all case cells and all control cells without distinguishing between cell types). For astrocytes, we identified 33 exons (10 with |ΔΨ| ≥ 20%) whose disease-associated splicing dysregulation was only observable in astrocytes and not in pseudobulk. By definition, using pseudobulk instead of astrocytes can only increase read numbers and statistical power. However, if non-astrocytic reads show opposite behavior between cases and controls or lack substantial case-control changes while outnumbering astrocytic reads, an exon’s significance in astrocytes may be masked in pseudobulk data (Figure 3E). Indeed, this is the case for the solute carrier family 25 member 26 (*SLC25A26*) gene, where the relatively strong 31% ΔΨ in astrocytes is obscured by a near-zero, non-significant ΔΨ in oligodendrocytes (Figure S9A). Likewise, we found 13 exons whose inclusion differences between cases and controls could only be observed in oligodendrocytes but not in pseudobulk. The centrosomal protein 97 (*CEP97*) gene illustrates this behavior for oligodendrocytes (Figure S9B). Overall, we found 18% of significant exons for excitatory neurons (95% confidence interval: [13%, 23%]) to be non-observable in pseudobulk and 15% for inhibitory neurons (95% confidence interval: [6%, 24%]). For astrocytes and oligodendrocytes, we found 29% (95% confidence interval: [21%, 38%]) and 25% (95% confidence interval: [13%, 37%]), respectively (Figure 3F). In summary, while still in the minority, a substantial number of exons can only be observed when testing in specific cell types rather than in pseudobulk.

### Splice-donor and -acceptor site dysregulation

FTD samples also exhibited changes in splice-site usage. Using an approach that takes into account the effect of the ONT error rate on splice-site mapping accuracy,^43,44^ we identified 16 dysregulated splice-donor sites in excitatory neurons, 22 in inhibitory neurons, 15 in astrocytes, 23 in oligodendrocytes, and 18 in microglia. Looking at splice-acceptor sites, we found 17 to be dysregulated in excitatory neurons, 11 in inhibitory neurons, 7 in astrocytes, 12 in oligodendrocytes, and 10 in microglia (Figure 3G). Here, Ψ was defined as the fraction of reads using the most common splice site out of the total number of reads using either of the two most common splice sites, and an FDR of 0.05 and ΔΨ ≥ 10% were required for a splice site to be considered significantly dysregulated. Notably, there was no overlap between the genes affected by donor-site dysregulation and those affected by acceptor-site dysregulation in any of the major cell types.

### Cell-type-specific dysregulation patterns

**Figure 4.**
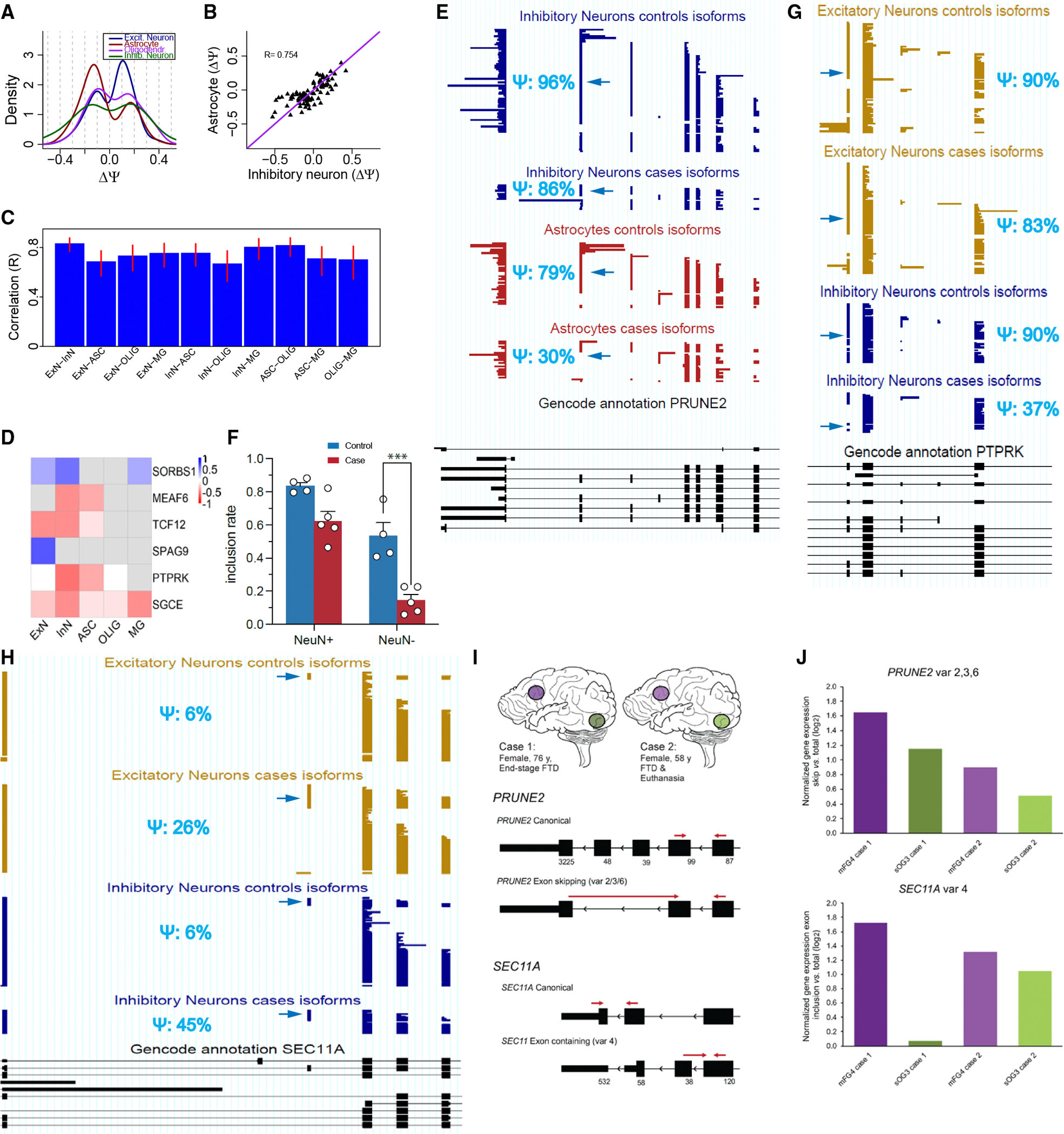
Cell-type specificity of FTD-associated splicing dysregulation (A) ΔΨ distribution of significant exons with FDR ≤ 0.05 for excitatory neurons, astrocytes, oligodendrocytes, and inhibitory neurons. (B) Dot plot of ΔΨ values in inhibitory neurons and astrocytes. (C) Bar plot of correlations of ΔΨ values for pairs of cell types. Error bars indicate 95% confidence intervals. (D) Heatmap showing ΔΨ values for the exons with |ΔΨ| ≥ 45% and the genes they belong to. (E) Inhibitory neuron (top two tracks in blue) and astrocyte data (red) for *PRUNE2*. Each line corresponds to one molecule. Reads from all case samples are grouped together; reads from all control samples are grouped together. Only informative reads for the highlighted exon are shown. Bottom (black) track: GENCODE annotation (v.34) for *PRUNE2*. (F) Validation of expression of *PRUNE2* alternative exons. Quantitative analysis of inclusion rate of exons 17 and 18 by normalizing with the expression of exons 15 and 16 in NeuN^+^ and NeuN^−^ cells from healthy (control) and FTD (case) samples. *n* = 4 for controls and *n* = 5 for cases. Statistical significance was calculated by two-way ANOVA, followed by Tukey test for multiple comparisons. ***p < 0.001. Error bars indicate SEM. (G) Excitatory neuron (top tracks in light brown) and inhibitory neuron data (bottom tracks in blue) for *PTPRK*. Each line corresponds to one molecule. Reads from all case samples are grouped together; reads from all control samples are grouped together. Only informative reads for the highlighted exon are shown. Bottom (black) track: GENCODE annotation (v.34) for *PTPRK*. (H) Excitatory neuron (top tracks in light brown) and inhibitory neuron data (bottom tracks in blue) for *SEC11A*. Each line corresponds to one molecule. Reads from all case samples are grouped together; reads from all control samples are grouped together. Only informative reads for the highlighted exon are shown. Bottom (black) track: GENCODE annotation (v.34) for *SEC11A*. (I) Experimental setup (top) for RT-qPCR analysis of splicing events for left middle frontal gyrus (purple) vs. left superior occipital gyrus (green) in two patients with GRN-FTD. Overview of *PRUNE2* (middle) and *SEC11A* (bottom) alternatively spliced exons and splicing-specific qPCR primer design. Numbers below exons indicate their size in nucleotides. (J) Validation of expression of *PRUNE2* (top) and *SEC11A* (bottom) alternative exons in differentially affected brain areas from independent GRN-FTD samples. Quantitative analysis of the FTD-specific exclusion rate of exons 17 and 18 (*PRUNE2*) and inclusion rate of exon 5 (*SEC11A*) was performed by normalizing the expression level of the alternatively spliced transcript to that of the canonical transcript (*PRUNE2*: exons 15 and 16; *SEC11A*; exons 6 and 7). For both genes, a higher proportion of the FTD-specific splicing events was detected in the more affected mFG4 vs. sOG3 area in two patients with GRN-FTD (log_2_FC_frontal vs. occipital_ *PRUNE2* case 1: 0.50, *PRUNE2* case 2: 0.39; *SEC11A* case 1: 1.65; *SEC11A* case 2: 0.27). Disease progression seemed to correlate with rate of splicing since, for both genes, a higher proportion of the FTD-specific splicing events was detected in GRN-FTD case 1, independent of brain region.

We then investigated to what extent the FTD-associated splicing changes were similar across cell types. For significant exons, the ΔΨ distribution is expected to show aspects of bimodality, as significance can be achieved more easily when |ΔΨ| is higher. Astrocytes showed a strong trend toward more skipping in the disease state, while excitatory neurons were, in contrast, prone to more inclusion, whereas dysregulation events among inhibitory neurons and oligodendrocytes were more evenly distributed between increased inclusion and skipping (Figure 4A). When splicing changes were quantifiable in multiple cell types, we observed a significant correlation. For example, ΔΨ values showed a Pearson correlation of 0.754 between inhibitory neurons and astrocytes (95% confidence interval: [0.651, 0.829]), although with a few notable exceptions (Figure 4B). Interestingly, ΔΨ values correlated highly between excitatory and inhibitory neurons for exons that were significant in at least one of the five major cell types, with astrocytes and oligodendrocytes demonstrating the second-highest correlation (Figure 4C). Thus, neuronal splicing dysregulation in FTD appears to differ from that of non-neuronal cell types.

We then considered exons with the highest splicing changes (|ΔΨ| ≥ 45% in at least one cell type). Six exons from six different genes showed such a marked difference in exon inclusion. In agreement with the above observations, these exons showed a tendency for disease-associated splicing changes in only a few cell types: sometimes because of low expression in all other cell types and sometimes due to the quantifiable absence of disease-associated changes in other cell types. Thus, these very strong cell-type-specific splicing changes often occurred in a cell-type-restricted manner (Figure 4D).

An example of astrocyte-specific FTD-associated exon skipping was found in the synaptic gene prune homolog 2 with BCH domain (*PRUNE2*). In inhibitory neurons, an exon shifts from a Ψ of 96% in controls to 86% in FTD samples, while in astrocytes, the drop is much more substantial, from 79% in controls to 30% in FTD samples (Figure 4E). The behavior in excitatory neurons was similar to what was seen with inhibitory neurons (Figure S10). While the strong astrocytic dysregulation causes this exon’s shift to be observable in pseudobulk, the fact that its dysregulation occurs predominantly in astrocytes and not neurons would not have been revealed by bulk-seq methods. Despite a 0.09 FDR in astrocytes, this observation was validated by RT-sqPCR. Sorted NeuN^+^ cells showed a weak FTD-specific reduction in inclusion of the abovementioned *PRUNE2* exon and an adjacent exon that was strongly coordinated with it, but a marked decrease in inclusion for those exons in NeuN^−^ glial cells (Figures 4F, S8A, and S8B). Additional validations of neuron-specific, glia-specific, and broad splicing-dysregulation events were mostly consistent with our calculated values (Figures S8C–S8I). In total, 7 out of 9 tested exons conformed with the trends seen in our sequencing-based results. Another example of cell-type-specific FTD-associated splicing dysregulation was found in the protein tyrosine phosphatase receptor type K (*PTPRK*) gene. A slight disease-associated reduction in exon inclusion of −7% (90% in controls and 83% in cases) was observed in excitatory neurons, whereas a much stronger drop of −53% (90% in controls and 37% in cases) was seen in inhibitory neurons. Thus, neuronal subtypes also differ in their FTD-associated splicing dysregulation (Figure 4G). Notably, in *PRUNE2*, we observe the disease-associated inclusion of a cryptic exon in the same intron where an FTD-associated cryptic exon was observed^7,12^ (Figure S11). In the bridging integrator 1 (*BIN1*) gene, intra-glial differences can be seen, where astrocytes exhibit a ΔΨ of 37% while oligodendrocytes display a smaller ΔΨ of 8% (Figure S12).

### FTD-affected brain regions show enhanced splicing events

As FTD is a progressive disease, beginning in the frontal and temporal lobes and gradually spreading laterally and posteriorly, we investigated whether frontal regions exhibited stronger FTD-specific splicing dysregulation than posterior regions in two additional GRN-FTD samples using quantitative real-time reverse-transcription PCR (RT-qPCR) on whole-tissue gray matter. We measured the following genes: (1) *PRUNE2*, because it has a high ΔΨ value, entails skipping of two exons, and is astrocyte specific (Figures 4I and 4J); (2) *SEC11A*, because its exon inclusion is neuron specific with a moderate ΔΨ (Figures 4H–4J); and (3) *CD47*, which shows skipping of two exons and significant splicing changes in most cell types (Figures S13A–S13C). In addition, the *SEC11A* exon and one of the *CD47* exons are frameshifting. Two patients with GRN-FTD were selected from the Netherlands Brain Bank for whom both the medial frontal gyrus 4 (mFG4) and the superior occipital gyrus 3 (sOG3) were available (Figure 4I). Case 1 was an end-stage patient with FTD, with high atrophy in the frontal region and an apparently spared occipital region, whereas case 2 was a patient with FTD who died from euthanasia and had moderate atrophy in the frontal cortex and no atrophy in the occipital cortex (Table S3). Case 2 was previously described (as family 4, patient III:1).^45^

Quantitative expression analysis of the splicing events in the mFG4 and sOG3 of the two cases was performed by normalizing the expression of transcripts with or without the FTD-specific exons with the expression level of the canonical transcript to compensate for regional differences in expression level. For both subjects, we were able to validate a higher expression of the FTD-specific splicing event—be it more skipping or more inclusion—in the more-affected mFG4 region compared to the less-affected or unaffected sOG3 region for *PRUNE2* and *SEC11A* (Figure 4J). The more severe case 1 generally showed a higher expression level of transcripts that were associated with FTD in our sequencing data than case 2, as well as a higher region-specific proportion of these dysregulated splicing events, i.e., they were seen more frequently in the frontal region than in the occipital region (log_2_ fold change [log_2_FC]_frontal vs. occipital_; Figure 4J). The alternative splicing events of *PRUNE2* and *SEC11A* result in proteins with premature stop codons and, hence, proteins that have a (shorter) C terminus with a different protein sequence.

Alternative splicing of two exons from the *CD47* gene resulted in a higher expression of *CD47* variant 2 (lacking exons 9 and 10) in the more affected mFG4 vs. the sOG3 (Figure S13C). Notably, the *CD47* variant 3 transcript (lacking exon 10 only) did not show this type of regulation; this could reflect region-specific alternative-exon usage. Transcript variant 2, the FTD-specific variant in both our sequencing and RT-qPCR data, results in a premature stop codon, and as a result, the protein is the shortest form of the three variants at the C terminus.

In addition, we tested whether the cryptic exon in *UNC13A* that has been linked to ALS and FTLD-TDP,^7^ which lies between exons 20 and 21, is differentially included in the mFG4 vs. sOG3 regions. Inclusion of this cryptic exon is dependent on mislocalization of TDP-43 and is potentiated by intronic risk-associated single-nucleotide polymorphisms (SNPs).^7^ Transcripts with this exon encode a premature stop codon and are targets for nonsense-mediated decay of mRNA. In line with this, detection of the cryptic transcript from total RNA was low but feasible. As with the other genes we tested, *UNC13A* showed a high region-specific profile for inclusion of this cryptic exon, with the more affected mFG4 region having a higher expression of transcripts containing the cryptic exon than the less affected sOG3 area (log_2_FC_frontal vs. occipital_; Figure S13D) for both patients. Our single-cell, long-read analysis detected only a handful of reads that supported splice sites on one or both sides of this cryptic exon—not enough for us to calculate ΔΨ values in either a bulk or a cell-type-specific manner.

### Splicing dysregulation in excitatory subtypes associated with distinct cortical layers

The observation that FTD-associated splicing dysregulation was obscured in pseudobulk but apparent when considering cell-type-specific isoforms prompted us to further investigate subtypes of excitatory neurons. We found multiple distinct clusters of excitatory neurons (Figure S14). For splicing-dysregulation analysis, we restricted our analysis to the three largest groupings to achieve adequate statistical power: the cluster marked by RAR-related orphan receptor B (*RORB*), the cluster marked by semaphorin 3E (*SEMA3E*), and the two somewhat connected clusters marked by cut-like homeobox 2 (*CUX2*). Each of these is generally associated with certain cortical layers, with *CUX2*-marked neurons corresponding to layers L2–3,^46–48^ *RORB*-marked neurons corresponding to layers L3–5, and *SEMA3E*-marked neurons corresponding to layers L4–6 (Figure S14). We first tested for differential exon inclusion in FTD cases and controls in *CUX2*-marked L2–3 excitatory neurons and found 33 exons with the same requirements as previously for cell-type-specific exons (FDR of 0.05 and |ΔΨ| ≥ 20%, two-thirds of samples in each group contributing reads, and two-thirds of sample ΔΨ values in the “correct” order compared to case and control ΔΨ values) (Figure S15A). *RORB*-marked L3–5 excitatory neurons had 16 such dysregulated exons, and *SEMA3E*-marked L4–6 excitatory neurons had 11. Notably, TDP-43 pathology in patients with *GRN* mutations was shown to be consistently observed in upper layers (L2–3), with relatively less involvement of the deeper cortex (L4–6) across FTLD-TDP cases,^49^ supporting a link between layer-specific splicing dysregulation and the extent of TDP-43 pathology. In our case, as a proportion of cell count, both the highest and deepest layers had more splicing dysregulation events than the middle layers (Figure S16). When considering exons that demonstrated significant FTD-associated dysregulation in excitatory neurons of all subtypes, we found significant correlations between the subtype-specific ΔΨ values. Indeed, *SEMA3E*-marked L4–6 excitatory neurons and *CUX2*-marked L2–3 excitatory neurons often showed similar ΔΨ values between cases and controls, though with marked exceptions (Figure S15B).

We then focused on exons that did not pass the significance threshold of FDR = 0.05 for differential exon inclusion in FTD samples vs. controls at the level of all excitatory neurons but did pass this threshold in at least one of the layer-specific subtypes. As expected, these exons’ ΔΨ values were not correlated between *SEMA3E*-marked L4–6 excitatory neurons and *CUX2*-marked L2–3 excitatory neurons. Thus, even for excitatory-neuron subtypes from different layers, splicing dysregulation in one subtype can be masked by that of other subtypes (Figure S15C). Comparing the three layer-specific clusters of excitatory neurons in terms of their splicing dysregulation, we observed closer clustering of the upper layers, L2–3 and L3–5, with L4–6 excitatory neurons being the outgroup (Figure S15D). Thus, distinct FTD-associated dysregulation could be found in different cortical layers. In *CUX2*-marked L2–3 excitatory cells, 20% (95% confidence interval: [12%, 29%]) of significant exons would have been missed if excitatory neurons were only analyzed as a whole, without consideration of subtypes—the highest proportion among the three major excitatory subtypes (Figure S15E). *TCF12* is one example of varied levels of dysregulation among the excitatory subtypes associated with distinct cortical layers, with all showing increased skipping in FTD of an alternative exon, but with *SEMA3E*-marked L4–6 cells demonstrating the strongest ΔΨ, while *RORB*-marked L3–5 cells and *CUX2*-marked L2–3 exhibited case-control differences of smaller magnitude (Figure S15F).

## DISCUSSION

Here, we present the first case-control, isoform-resolved study of the human brain at single-cell resolution for a neurological disease. Thus, in one assay, we derive a short-read, single-cell view of gene expression dysregulation, as well as a long-read, splice-junction-enriched view of splicing dysregulation in GRN-FTD. Using the short-read data, we found that neurons and OPCs exhibited more down-regulated genes than up-regulated genes, but the reverse was true for astrocytes and oligodendrocytes. We also observed strong enrichment of synaptic terms in dysregulated genes, especially in excitatory neurons, but also in inhibitory neurons, astrocytes, and OPCs, suggesting alterations of synaptic connections in GRN-FTD. We therefore focused our long-read approach on 3,630 genes, including genes with FTD- and neurodegeneration-associated splicing dysregulation and synaptic genes. This enabled an in-depth characterization of these genes in all major cell types. We found that exons whose inclusion is up-regulated in GRN-FTD samples differ from those that are down-regulated. Namely, the former have a median size of 95 bp, while the latter have a median size of 57 bp. Given the distinct splicing regulation of short exons, such as for example microexons,^40^ our findings suggest that distinct splicing programs are affected in GRN-FTD.

Interestingly, splicing dysregulation is associated with dysregulation of gene expression levels in inhibitory neurons. This suggests a role for stoichiometry in cell-type-specific splicing dysregulation or a role for linked transcription and splicing^50,51^ in the disease. Regardless of whether either model or both are correct, the connected dysregulation of these two processes strongly advocates for methods, like the one we use here, that interrogate both phenomena—in FTD and possibly in other forms of neurodegeneration. Given that transcript-level and splicing dysregulation are linked and that the promoter sequence can influence splicing,^52^ another question remains: is the decision to produce splicing dysregulation in GRN-FTD made at the promoter? Answering this question would require a dedicated experimental investigation that is beyond the scope of this work.

Surprisingly, our results indicate that the largest disease-related splicing changes (|ΔΨ| ≥ 45%) occur either in only a few cell types or in genes with restricted expression patterns. On the other hand, when a gene is widely expressed across multiple cell types and splicing dysregulation is more modest, we find a correlation between cell types. Notably, neuronal subtypes show more similar exon-usage changes in GRN-FTD than any other pair of cell types, indicating distinct dysregulation of glial and neuronal splicing in GRN-FTD. Exons for which dysregulation is strong in one cell type but weak to absent in other cell types are of specific interest (Table S2), as they highlight the importance of cell-type-specific alternative splicing that may be relevant to future diagnostic or therapeutic strategies. Even when analyzing bulk tissue (gray matter), several of these cell-specific splicing events could be detected by RT-qPCR (*PRUNE2* and *SEC11A*). Regarding intervention, the community should consider the possibility that a correction in one cell type might cause problems in another, as the “wrong” isoform in one cell type could be the “correct” isoform in another—as seen, for instance, in an exon of *TCF12*, where neurons in FTD exhibit the low inclusion rate that is seen in both FTD and control astrocytes. It would also be interesting to determine, via future studies, the extent to which the splicing changes we have identified here are present in other forms of FTD or ALS with TDP-43 pathology, such as those with *C9ORF72* repeat expansions.

On the other hand, we were able to correlate the level of these splicing events to that of brain atrophy in two other patients with GRN-FTD and showed that the more affected frontal cortex has a higher level of splicing events than the less affected occipital cortex. In addition, we observed that splicing was correlated with the stage of disease, as the less severe case 2 exhibited fewer of the FTD-associated isoforms than the more severe case 1. It is also worth noting that these changes could still be detected in case 2 despite the fact that the tissue used was from the patient’s less atrophied hemisphere. While it is possible that this approach could introduce the additional variable of brain-region differences into the equation, it provides an independent method of validation of our sequencing findings that conforms with the key regionally specific character of the disease. With this approach, we were also able to observe an association with GRN-FTD pathology for the previously described^7^ *UNC13A* cryptic exon that has been linked to TDP-43 depletion; however, we did not have sufficient reads to calculate a case-control difference in our sequencing analysis.

Taken together, our strategy reveals the cell-type-specific basis of splicing and gene expression dysregulation in GRN-FTD and provides a means to shed further light on all neurodegenerative diseases.

### Limitations of the study

Given that this was a postmortem study, it was of course impossible to characterize the splicing profiles of cells that were already lost. These may have been distributed unequally among cell types due to selective vulnerability, which has, for instance, been observed in AD.^53^ Elucidating cell-type-specific splicing patterns of cells lost early on is therefore a potentially valuable approach that would require further research. In addition, an analysis of this kind would benefit from larger sample sizes, both in terms of the number of individuals studied and the number of reads sequenced per individual. The challenge of obtaining postmortem human samples limits the former; the latter may require improvements in method or new approaches. In this study, we made use of several distinct definitions of splicing dysregulation—with the most stringent imposing requirements based on individual variation and sample age—to address this limitation while avoiding the loss of potentially valuable signals.

## RESOURCE AVAILABILITY

### Lead contact

Requests for further information and resources should be directed to and will be fulfilled by the lead contact, Hagen U. Tilgner (hagen.u.tilgner@gmail.com).

### Materials availability

This study did not generate new unique reagents.

### Data and code availability

- Gene expression data from short-read sequencing have been deposited at the NCBI Gene Expression Omnibus (GEO) database with accession number GEO: GSE250280.
- Long-read sequencing data have been deposited at the NCBI Sequence Read Archive (SRA) database with accession number SRA: PRJNA1238317.
- All other data reported in this paper will be shared by the lead contact upon request.
- This paper does not report original code.
- Any additional information required to reanalyze the data reported in this paper is available from the lead contact upon request.

## STAR★METHODS

### EXPERIMENTAL MODEL AND STUDY PARTICIPANT DETAILS

Superior frontal gyrus tissues from 6 controls and 6 FTD human donors were obtained from tissue banks maintained by the Neurodegenerative Disease Brain Bank at the University of California, San Francisco, according to institutional review board–approved protocols.^57^ Tissues were flash-frozen and kept at −80°C until processing. Additional information about the donors can be found in Table S1. In addition, tissue blocks from two separate human donors with FTD were obtained from the Netherlands Brain Bank for use in qPCR validation (more information in the Quantitative real-time PCR section below).

### METHOD DETAILS

#### Single-nucleus isolation

The single-nucleus suspension was isolated from fresh-frozen human brain samples from a previous protocol with modifications.^58,59^ All procedures were done on ice or at 4°C. In brief, ~30 mg of frozen tissue per sample was dissected in a sterile dish on dry ice and transferred to 1.5 mL of nuclei pure lysis buffer (MilliporeSigma, L9286) and homogenized with a Dounce tissue grinder (Sigma, D8938–1SET) with 20 strokes with pestle A and 15 strokes with pestle B. The nucleus suspension was filtered by loading through a 35-μm-diameter filter and followed by centrifugating for 5 min at 600×g and 4°C. The nuclei pellet was collected and washed three times with cold wash buffer, which consisted of the following reagents: 1× PBS (Thermo Fisher Scientific, 70011044), 20 mM DTT (Thermo Fisher Scientific, P2325), 1% BSA (Thermo Fisher Scientific, 37525) and 0.2 U/μL of RNase inhibitor (Ambion, AM2682). After removing the supernatant from the last wash, nuclei were resuspended in 1 mL of 0.5 μg/mL of DAPI (Sigma, D9542) containing wash buffer to stain for 15 min. The nuclei were passed through a 35 μm strainer and then sorted using the Sony MA900 sorter with FlowJo version 10 software (Figure S17). These were collected by centrifugation at 600*g* for 5 min at 4°C and then resuspended in wash buffer to reach a final concentration of 1 × 10^6^ nuclei per milliliter after counting with DAPI using a Countess II cell counter (Thermo Fisher Scientific, A27977).

#### 10× Genomics 3′ library construction and short-read sequencing

10× Genomics 3′ library construction was prepared with Chromium Single Cell 3′ Reagent Kits v3.1 (10× Genomics, PN-1000268) following the manufacturer’s instructions with single-nucleus suspension obtained from the last step. 10× Genomics 3′ libraries were loaded on an Illumina NovaSeq 6000 with PE 2 × 50 paired-end kits by setting the read length as followings: 28 cycles for Read1, 8 cycles for i7 index and 91 cycles for Read2.

#### Linear PCR and exome enrichment (LAP-CAP)

##### Linear/asymmetric PCR steps to remove non-barcoded cDNA

The first round PCR protocol (95°C for 3 min, 12 cycles of 98°C for 20 s, 64°C for 30 s and 72°C for 60 s) was performed by applying 12 cycles of linear/asymmetric amplification to enrich molecules containing 10× barcode (30 ng cDNA generated by using 10× Genomics Chromium Single Cell 3ʹ GEM kit) with primer “Partial Read1,” then the product was purified with 0.8× SPRIselect beads (Beckman Coulter, B23318) and washed twice with 80% ethanol. The second-round PCR was performed by applying 6 cycles of exponential amplification under the same conditions with forward primer “Partial Read1” and reverse primer “Partial TSO,” then the product was purified with 0.6× SPRIselect beads and washed twice with 80% ethanol, and finally eluted in 30 μL buffer EB (Qiagen, 19086). KAPA HiFi HotStart PCR Ready Mix (Roche, KK2601) was used as polymerase for all the PCR amplification steps in this paper except for the 10× Genomics 3′ library construction. QC was performed using Genomic DNA ScreenTape and reagents (Agilent, 5067–5365, 5067–5366) on Agilent TapeStation.

##### Exome capture to enrich for spliced cDNA

Exome enrichment was applied to the cDNA purified from the previous step by using customized probe set described in probe design section (SureSelect Custom Tier4, 16Rxns, Agilent, 5191–6915) and the reagent kit SureSelectXT HSQ (Agilent, G9611A) according to the manufacturer’s manual. First, the block oligo mix was made by mixing equal amount (1 μL of each per reaction) of primers Partial Read1 and Partial TSO (sequences shown above) with the concentration of 200 ng/μL (IDT), resulting in 100 ng/μL. Next, 5 μL of 100 ng/μL cDNA diluted from the previous step was combined with 2 μL block mix and 2 μL nuclease free water (Invitrogen, AM9937), then the cDNA-block oligo mix was incubated on a thermocycler under the following condition to allow block oligo mix to bind to 5′ and 3′ end of the cDNA molecule: 95°C for 5 min, 65°C for 5 min, 65°C on hold. For the next step, the hybridization mix was prepared by combining 20 mL SureSelect Hyb1, 0.8 mL SureSelect Hyb2, 8.0 mL SureSelect Hyb3, and 10.4 mL SureSelect Hyb4 and kept at room temperature. Once the reaction reached to 65°C on hold, 5 μL of probe, 1.5 μL of nuclease free water, 0.5 μL of 1:4 diluted RNase Block and 13 μL of the hybridization mix were added to the cDNA-block oligo mix and incubated for 16–24 h at 65°C. When the incubation reached the end, the hybridization reaction was transferred to room temperature. Simultaneously, an aliquot of 75 μL M-270 Streptavidin Dynabeads (Thermo Fisher Scientific, 65305) prepared by washing three times and resuspending with 200 μL binding buffer. Next, the hybridization reaction was mixed with all the M270 Dynabeads and placed on a Hula mixer with low speed for 30 min at room temperature. During the incubation, 600 μL of wash buffer 2 (WB2) was transferred to 3 wells of 0.2 mL PCR tube and incubated at 65°C. After the 30 min incubation, the buffer was replaced with 200 μL of wash buffer 1 (WB1). Then the tube containing hybridization product bound to M-270 Dynabeads was put back to the Hula mixer for another 15 min incubation with low speed. Next, the WB1 was replaced with WB2 and the tube was transferred to the thermocycler for the next round of incubation. Overall, the hybridization product bound to M-270 Dynabeads was incubated in WB2 for 30 min at 65°C, and the buffer was replaced with fresh pre-heated WB2 every 10 min. When the incubation was over, all liquid was removed and the beads were resuspended in 18 μL of nuclease-free water and stored at 4°C. Next, the spliced cDNA which bound with the M-270 Dynabeads was amplified with primers Partial Read1 and Partial TSO (10 ng/μL) by using the following PCR protocol: 95°C for 3 min, 12 cycles of 98°C for 20 s, 64°C for 60 s and 72°C for 3 min. The amplified targeted cDNA was isolated from M-270 beads as supernatant and then purified with 0.6× SPRIselect beads.

##### Long-read library prep and sequencing

For each sample, ~75 fmol cDNA processed through LAP-CAP underwent ONT library construction by using Ligation Sequencing Kit (Oxford Nanopore Technologies, SQK-LSK110) according to the manufacturer’s protocol (Nanopore Protocol, Amplicons by Ligation, Version: ACDE_9110_v110_revC_10Nov2020). The ONT library was loaded to PromethION Flow Cell (Oxford Nanopore Technologies, FLO-PRO002) and sequenced with a PromethION sequencer for 72 h. Base-calling was performed with Guppy by setting base-quality score >7.

##### Semi-quantitative reverse-transcription PCR (RT-sqPCR)

After isolation using the same method as for snRNAseq, nuclei were stained with NeuN Alexa Fluor 488 (1:1000; Millipore Sigma, MAB377) and incubated overnight at 4°C. 200k NeuN-positive and 200k NeuN-negative nuclei were sorted with BD FACSAria II cell sorter and pelleted at 600×g for 5 min in wash buffer. Total or sorted nuclei were homogenized by vortexing for 1 min in RLT buffer with 1% β-mercaptoethanol. RNA was isolated with the RNeasy Plus Micro Kit (Qiagen, Cat# 74034) by following the manufacturer’s protocol, and the remaining DNA was removed by gDNA Eliminator columns. Purified mRNA was then converted to cDNA with the PrimeScript RT Master Mix Kit (Takara, Cat# RR036A). RT-sqPCR was performed in triplicate on the CFX384 Touch Real-Time PCR Detection System (Bio-Rad) with TB Green Premix Ex Taq II master mix (Takara, Cat# RR820A). Quantification of agarose gel images was performed using Image Lab software (Bio-Rad) (Figure S8B).

##### Quantitative real-time reverse-transcription PCR (RT-qPCR)

Tissue blocks of two FTD cases with verified GRN mutations were obtained from the Netherlands Brain Bank (NBB, Amsterdam, The Netherlands) (Table S3). For each subject, at least 2 individual rounds of cryo-sectioning, RNA isolation and cDNA synthesis were performed. In addition, for each subject and cDNA synthesis, qPCRs were carried out in duplicate by two individual researchers. Data were collected and the mean of all values is presented.

###### Tissue collection.

Cryo-sections (25 μm, total ~10–30 mg) were cut from tissue blocks in the cryostat (−15°C), taking gray matter only.

###### RNA isolation.

Tissue was lysed in 700 μL Trizol Reagent (Thermo Fisher, AM9738) according to the manufacturer’s protocol; after addition of chloroform, the sample was shaken and centrifuged (15 min 20,000×g) to get rid of proteins and genomic DNA. The RNA-containing upper phase was transferred to a Qiagen mini column, and RNA was isolated with the RNeasy Plus Mini Kit (Qiagen, 74134) by following the manufacturer’s protocol.

###### cDNA synthesis.

Random-primed (25 pmol; Eurogentec, Belgium) cDNA synthesis was performed on individual RNA samples (~200–400 ng total RNA) using MMLV reverse transcriptase (Promega, M1701).

###### Real-time qPCR.

Real-time qPCR reactions (7 μL; Applied Biosystems, QuantStudio 5 Real-Time PCR System, A28140) were performed using a 384-well format with transcript-specific primers (300 nM) on cDNA corresponding to ~0.4 ng RNA) and SYBR Green reagents (Meridian Bioscience, SensiFAST SYBR Hi-ROX Kit, BIO-92020), and relative gene expression calculations were performed as described previously.^60^ The geometric means of expression levels of four housekeeping genes (*GAPDH*, *RPLP0*, *TRFC*, *HPRT*) were used as input controls. Housekeeping gene-normalized expression values of transcripts containing skipped exons (*PRUNE2*, *CD47*), or included exons (*SEC11A*) were expressed as a log_2_-ratio vs. either total transcript (*PRUNE2*, *SEC11A*), or vs. transcript with all exons in (*CD47*) to correct for differential expression by tissue. To ensure clear graphical presentation, the expression values have been translocated on the y axis for some genes: *SEC11A* + 4; CD47 var3 +2; UNC13A CR +8. In addition, we report the inclusion/exclusion rate as the ratio between expression in the frontal versus occipital area as log_2_FC. For primer sequences, see key resources table.

### QUANTIFICATION AND STATISTICAL ANALYSIS

#### Data processing for single-cell short-read analysis

The 10× Cell Ranger pipeline (version 3.1.0) was run on raw Illumina sequencing data to obtain single-cell count matrices which were analyzed using Seurat v 4.1.0.^29^ For all 12 samples, nuclei that had gene counts range between a lower bound of 500 and an upper bound of 6500~9000, and <15% mitochondrial gene expression were kept. This yielded 6787, 9830, 10481, 8296, 8055 and 7486 nuclei for control1 ~ control6, 7384, 12100, 5539, 6340, 8704 and 8316 for case1 ~ case6. UMI numbers and mitochondrial gene expression percentages were regressed from each nucleus and the matrix was log normalized and scaled to 10,000 reads/cell. All 12 datasets were integrated by running Harmony^56^ after performing PCA. Next, we clustered cells using the Louvain algorithm, setting the resolution parameter to 0.6. UMAP non-linear dimensionality reduction was performed on the harmony-integrated data.

#### Cell types and subtypes identification

Major cell types were assigned by identifying canonical marker genes for each cluster.^47,61–63^ Cell subtypes were assigned based on cluster-specific markers identified among top 20 hits with FindMarkers function of Seurat 4.1.0. The corresponding cortical layers of excitatory neuron subtypes were assigned according to representative layer specific markers indicated in previous studies^46–48^ (Figure S15).

#### Differential gene expression calling from short reads

Differential expressed gene list of each major cell type were identified by comparing case and control group with MAST,^33^ after imposing cutoffs of |log_2_FC| ≥ 0.2 and adjusted *p*-value ≤0.05.

#### GO-enrichment analysis for differentially expressed genes

GO-enrichment analysis was performed by running clusterProfiler_4.2.2^34^ (pAdjustMethod = “BH”, pvalueCutoff = 0.1). For each major cell type, the differentially expressed genes that met the criterion of adjusted *p*-value ≤0.05 and |log_2_FC| > 0.2 derived from the step mentioned before were taken as the query list, and all the genes with |log_2_FC| > 0 in the corresponding cell type were taken as the background list.

#### Exome enrichment probe design

A list of genes including TDP-43 binding targets,^35^ synaptic genes,^36^ genes with highly variable exons,^28^ and genes associated with mis-splicing in AD,^37^ ALS,^16^ autism spectrum disorder,^38–40^ and schizophrenia^41^ was assembled. Using the GENCODE human annotation (release 34),^64^ all protein-coding transcripts of these genes were identified. For each exon–exon junction present in at least one transcript, 140 bases spanning the junction were selected, with 70 exonic bases on either side. If an exon was shorter than 70 bases, nucleotides from adjacent exon(s) were used until a length of 70 was reached. A 130-base minimum length was used when 140 bases were not available due to proximity to the beginning or end of a transcript; all sequences shorter than 130 bases were discarded. Sequences mapping to more than 5 locations in the genome and genes with fewer than 5 assigned probes were also discarded. A 120-mer was chosen from within the initial (130- to 140-base) sequence using Agilent Technologies’ method for maximizing hybridization efficiency.

#### Long-read mapping

The short-read cell-type assignments were used, via the single-nucleus barcodes, to determine the cell types of the long reads, using the GetBarcodes function of scisorseqr (version 0.1.6).^26^ Long reads were then mapped to the human genome (GRCh38) using Minimap2 (version 2.24),^54^ enabling the assignment of a gene and exon/intron chain to each read. Additionally, the closest published TSS and poly-A site within 50 bp of the 5′ and 3′ end, respectively, of the read were identified wherever possible with scisorseqr as previously described.^26^

#### Differential exon expression analysis and Ψ calculation

To account for the higher error rate of nanopore sequencing compared to short-read sequencing, and to mitigate the consequent distortions in unique molecular identifiers (UMIs), reads containing UMIs that were less than a Levenshtein distance of 4 away from UMIs that occurred more frequently were discarded. After this step, if a read had an intron chain that was not seen at least 5 times across the entire dataset of 12 samples, it was discarded.

Alternative exons were identified in the remaining long reads. An alternative exon was defined as an internal exon that was either entirely included or excluded from a transcript. For each qualifying exon, the number of inclusion and exclusion events was counted per cell type and condition (case or control). Inclusion events were defined as unique reads that either 1) included the entire exon and supported both of its splice sites or 2) started or ended within the exon and supported one of its splice sites. Exclusion events were defined as unique reads that span the region of the exon but include neither the exon nor ≥50 bases to either side of it. Total count was defined as the total number of reads overlapping the location of the exon (whether representing inclusion events, exclusion events, or neither).

Inclusion and exclusion counts were used to populate a 2 × 2 contingency table, whose statistical significance was assessed with Fisher’s exact test. If the table did not pass the chi-squared criterion, significance was not assessed. The false-discovery rate was calculated using the Benjamini–Yekutieli correction to account for multiple comparisons. Percent spliced in (Ψ) and ΔΨ were calculated as follows:

$$\Psi =\frac{\;\mathrm{number}\;\mathrm{of}\;\mathrm{inclusion}\;\mathrm{events}\;}{\;\mathrm{number}\;\mathrm{of}\;\mathrm{inclusion}\;\mathrm{events}\;+\;\mathrm{number}\;\mathrm{of}\;\mathrm{exclusion}\;\mathrm{events}}$$

$$\Delta \Psi =\Psi_{\mathrm{cases}\;}-\Psi_{\mathrm{controls}}$$

For an exon to be considered for analysis, it was required to have a Ψ that was ≥5% and ≤95%, and to have ≥10 reads per condition. In addition, the following requirement was enforced for an exon to be used in the final analysis:

$$\frac{\;\mathrm{number}\;\mathrm{of}\;\mathrm{inclusion}\;\mathrm{events+number}\;\mathrm{of}\;\mathrm{exclusion}\;\mathrm{events}\;}{\;\mathrm{total}\;\mathrm{count}\;}\geq 0.8$$

We previously included this calculation approach in the scisorATAC package^30^ as the casesVcontrols function.

#### Visualizing long reads

Long-read data was visualized using ScisorWiz (version 1.2.1.2).^55^ Only reads that overlapped the coordinates of the highlighted exon were included in the plot, unless otherwise noted. If more than 75 reads were present in a given condition, 75 reads were randomly selected to be plotted.

#### Comparing gene expression changes and splicing changes

To assess the relationship between alternative splicing and differential expression, the overlap between the set of significantly alternatively spliced genes (defined as those with at least one significantly dysregulated exon) and the set of significantly differentially expressed genes (defined as those with |log_2_FC| ≥ 0.1 and FDR ≤0.05) was assessed. Only genes that had been tested for both splicing and expression were considered. A 2 × 2 contingency table of the number that was significant by both measures, by only one measure, or by neither measure was created for each cell type and tested with Fisher’s exact test, and the corresponding odds ratio was calculated.

#### Quantifying alternative donor- and acceptor-site usage

After the initial steps of scisorseqr were run through the MapAndFilter step, sorted and index BAM files (with prefix “mapping.bestperRead.RNAdirection.withConsensIntrons”), separated by cell type, were used as input into IsoQuant.^44^ The resulting intron-count tables (files with suffix “intron_counts.tsv”) were then used to identify alternative donor and acceptor sites, calculate counts for each site, and sort them into groups relating to the same intron. For each group, a 2 × 2 contingency table was constructed with counts for cases and controls for the two sites with the highest total count across all samples. If there were no counts for either cases or controls, or if the total of all four cells of the table was less than 80% of the total count for the entire group, the group was discarded. Fisher’s exact test was used for each contingency table, with the Benjamini–Yekutieli used to account for multiple comparisons.

A splice-site version of Ψ was calculated as follows:

$$\Psi =\frac{\;\mathrm{count}\;\mathrm{for}\;\mathrm{top}\;\mathrm{site}\;}{\;\mathrm{count}\;\mathrm{for}\;\mathrm{top}\;\mathrm{two}\;\mathrm{sites}\;}$$

and the ΔΨ was again $\Psi_{\mathrm{cases}}-\Psi_{\mathrm{controls}}$.

## Supplementary Material

Supplementary Information

SUPPLEMENTAL INFORMATION

Supplemental information can be found online at https://doi.org/10.1016/j.celrep.2025.116198.

## Figures and Tables

**KEY RESOURCES TABLE:** 

REAGENT or RESOURCE	SOURCE	IDENTIFIER

Antibodies		

NeuN Alexa Fluor 488	Millipore Sigma	MAB377; RRID: AB_2298772

Biological samples		

Superior frontal gyrus tissue from 6 controls and 6 GRN-FTD human donors	Neurodegenerative Disease Brain Bank at University of California, San Francisco	P2503,P2805,P2943,P2843,P2920,P3040, P2921,P2937,P2942,P2947,P3006,P3041
Medial frontal gyrus (block 4, left) and superior occipital gyrus (block 3, l eft) tissue from 2 GRN-FTD human donors	The Netherlands Brain Bank, Amsterdam, The Netherlands	Case 1: NBB2021–101; Case 2: NBB2023–142; See Table S3 for detailed information

Chemicals, peptides, and recombinant proteins		

PBS	Thermo Fisher	70011044
DTT	Thermo Fisher	P2325
BSA	Thermo Fisher	37525
RNase inhibitor	Ambion	AM2682
DAPI	Sigma	D9542
EB buffer	Qiagen	19086
nuclease free water	Invitrogen	AM9937

Critical commercial assays		

Nuclei Isolation Kit	Millipore Sigma	L9286
Chromium Single Cell 3' Reagent Kits v3.1	10× Genomics	PN-1000268
KAPA HiFi HotStart PCR Ready Mix	Roche	KK2601
SPRIselect beads	Beckman	B23318
TapeStation DNA ScreenTape & Reagents	Agilent	5067-5365, 5067-5366
Qubit dsDNA HS Assay Kit	Invitrogen	Q32854
SureSelect Custom Tier4	Agilent	5191-6915
SureSelectXT HSQ	Agilent	G9611A
M-270 Streptavidin Dynabeads	Thermo Fisher	65305
Ligation Sequencing Kit	ONT	SQK-LSK110
PromethlON Flow Cell	ONT	FLO-PRO002
RNeasy Plus Micro Kit	Qiagen	74034
PrimeScript^™^ RT Master Mix Kit	Takara	RR036A
Ex Taq II master mix	Takara	RR820A

Deposited data		

Long-read (ONT) sequencing FASTQ files	NCBI’s Sequence Read Archive (SRA)	SRA: PRJNA1238317
Short-read (Illumina) sequencing FASTQ files	NCBI’s Gene Expression Omnibus (GEO)	GEO: GSE250280

Experimental models: Organisms/strains		

Human brain tissue	Neurodegenerative Disease Brain Bank at University of California, San Francisco	https://memory.ucsf.edu/research-trials/professional/neurodegenerative-disease-brain-bank
Human brain tissue	The Netherlands Brain Bank, Amsterdam, The Netherlands	https://www.brainbank.nl

Oligonucleotides		

Partial Read1	IDT	5' -CTACACGACGCTCTT CCGATCT-3 '
Partial TSO	IDT	5' -AAGCAGTGGTATCAACGCAGAGTACAT-3'
sqPCR *PRUNE2* alternative exon Fwd	IDT	5' -CTTGCTGTGACACGACCTTT-3 '
sqPCR *PRUNE2* alternative exon Rev	IDT	GCTCTCTGGAATGTGGATGC
sqPCR *PRUNE2* constant exon Fwd	IDT	GCTTATCAGAACTCAGTGGGC
sqPCR *PRUNE2* constant exon Rev	IDT	AGTTTTAGCTGCCTCTGATGC
sqPCR *GAPDH* Fwd	IDT	CCATCTTCCAGGAGCGAGAT
sqPCR *GAPDH* Rev	IDT	TGCTGATGATCTTGAGGCTG
*TCF12* alternative exon Fwd	IDT	TTGAGCAGCAACTTCACGAG
*TCF12* alternative exon Rev	IDT	AGGCAAACTGGTGGAAGGT
*TCF12* constant exon Fwd	IDT	GGACCATCCCATAATGCACC
*TCF12* constant exon Rev	IDT	TGGTTCAGGTCTGTGCTTGA
*C2CD5* alternative exon Fwd	IDT	GAGGAAATGCAGTTGTGGGG
*C2CD5* alternative exon Rev	IDT	AATTGGGCTGTTGTGAGTCG
*C2CD5* constant exon Fwd	IDT	AGGCAGTCATCATGTGGAGT
*C2CD5* constant exon Rev	IDT	ATTTGATGCCCTTGGTGTGC
*IFT88* alternative exon Fwd	IDT	TGGTCCAGAGATTGCAAAGTG
*IFT88* alternative exon Rev	IDT	CTTCTCTTTTGCCACGGGAG
*IFT88* constant exon Fwd	IDT	TGCCAGAAAACTGAAGAGGTTG
*IFT88* constant exon Rev	IDT	GGTCGTTCTATTTGAGGGCC
*DCUN1D2* alternative exon Fwd	IDT	ACCCAGGGCAGAAAGGTTTA
*DCUN1D2* alternative exon Rev	IDT	ATGGGACCCGCAGTAAAATG
*DCUN1D2* constant exon Fwd	IDT	GGCAGCAACTCAGTGTGAAT
*DCUN1D2* constant exon Rev	IDT	TAAACCTTTCTGCCCTGGGT
*CTTN* alternative exon Fwd	IDT	TCAAGGCAAAACGGAGAAGC
*CTTN* alternative exon Rev	IDT	TGGCCAGCTTCTCCTTGTAA
*CTTN* constant exon Fwd	IDT	AAAGGTTTCGGCGGCAAATA
*CTTN* constant exon Rev	IDT	TCTGTCTGTCTGCACACCAA
*RIMS1* alternative exon Fwd	IDT	AGAGCCCATGAATGTAGTTTGG
*RIMS1* alternative exon Rev	IDT	TGCTCGCGATCAAGTTCTTG
*RIMS1* constant exon Fwd	IDT	GCTTAGTAGTGGAGGAGCGA
*RIMS1* constant exon Rev	IDT	GGGAAATGGCGGAAACATCA
qPCR *SEC11A* Exon 4-Exon 5 junction Fwd^1,2^		5' -CCAGGGGATTTTATCAGGAGAAC-3'
qPCR *SEC11A* Exon 5 Rev^2^		AGGATCGTCACAATTCCAATATAAGG
qPCR *SEC11A* Exon 6 Fwd (Canonical)^2^		GGAATTGTGACGATCCTCATGAA
qPCR *SEC11A* Exon 7 Rev (Canonical)^2^		CAGGCTTCTTACTCACGATGAAC
qPCR *PRUNE2* Exon 15 Fwd^3^		CTTGCTGTGACACGACCTTTTAT
qPCR *PRUNE2* Exon 16-Exon 19 junction Rev^1,3^		ATATTGATGATGCTCTCTGGAATGT
qPCR *PRUNE2* Exon 15 Fwd (Canonical)^3^		TGCTGTGACACGACCTTTTATAA
qPCR *PRUNE2* Exon 16 Rev (Canonical)^3^		ATGATGCTCTCTGGAATGTGGAT
qPCR *CD47* Exon 10-Exon11 junction Fwd (variant 1)^1,4^		ATGATGAATAACTGAAGTGAAGTGATG
qPCR *CD47* Exon11 Rev (variant 1)^4^		GTTTCTTCTCCCCAACAGTGAAT
qPCR *CD47* Exon 8-Exon11 junction Fwd (variant 2)^1,4^		CCTAGGAATAACTGAAGTGAAGTGA
qPCR *CD47* Exon 11 Rev (variant 2)^4^		CTTCTCCCCAACAGTGAATCATC
qPCR *CD47* Exon 8 Fwd (variant 3)^4^		CCAATCAGAAGACTATACAACCT CC
qPCR *CD47* Exon 9-Exon 11 junction Rev (variant 3)^1,4^		TCCATCACTTCACTTCAGTTATTCAT
qPCR *UNC13A* CR-Exon21 junction Fwd^1,5^		GGATGGAGAGATGGAACCTGTT
qPCR *UNC13A* Exon21 Rev2		CTGGGCTGTCTCATCGTAGTAAA
qPCR *UNC13A* Exon19 Fwd (Canonical)^5^		TACAACCTGGACAAGCGAACT
qPCR *UNC13A* Exon20 Rev (Canonical)^5^		GCCTTTGATCTCCACACTGATG
qPCR *GAPDH* Exon 2 Fwd^6^		CACATCGCTCAGACACCATG
qPCR *GAPDH* Exon 3 Rev^6^		GCAACAATATCCACTTTACCAGAGTT
qPCR *RPLP0* Exon 6 Fwd^7^		TCTACAACCCTGAAGTGCTTGAT
qPCR *RPLP0* Exon 7 Rev^7^		CAATCTGCAGACAGACACTGG
qPCR *TFRC* Exon 17 Fwd^8^		CATTTGTGAGGGATCTGAACCA
qPCR *TFRC* Exon 18 Rev^8^		CGAGCAGAATACAGCCACTGTAA
qPCR *HPRT1* Exon 3 Fwd^9^		ATGGGAGGCCATCACATTGT
qPCR *HPRT1* Exon 3 Rev^9^		ATGTAATCCAGCAGGTCAGCAA

Software and algorithms		

scisorseqr	Joglekar et al.^26^	https://github.com/noush-joglekar/scisorseqr, v0.1.6
Minimap2	Li^54^	https://github.com/lh3/minimap2, v2.24
ScisorWiz	Stein et al.^55^	https://github.com/ans4013/ScisorWiz, vl.2.1.2
IsoQuant	Prjibelski et al.^44^	https://github.com/ablab/IsoQuant, v3.1.1
FlowJo	BD	v10
Guppy	ONT	v4.0.11
MinKNOW	ONT	v20.06.18
cellranger	10x Genomics	v3.1.0
Seurat	Hao et al.^29^	v4.1.0
Harmony	Korsunsky et al.^56^	V0.1.0
ClusterProfiler	Wu et al.^34^	v4.2.2
MAST	Finak et al.^33^	v1.20.0

## Highlights

- A case-control analysis of splicing dysregulation in GRN-FTD is presented
- Exons preferentially skipped in FTD are shorter than those that are up-regulated
- Up to 30% of cell-type-specific splicing changes are masked by other cell types
- In separate samples, frontal vs. occipital changes mirror FTD-associated changes
