# Supplementary material for: A single-cell, long-read, isoform-resolved case-control study of FTD reveals cell-type-specific and broad splicing dysregulation in human brain
Source: Cell Rep. Author manuscript; Available in PMC 2025 Oct 27. (PMC12558363; doi:10.1016/j.celrep.2025.116198)
Supplement: Supplementary Information [file NIHMS2110306-supplement-Supplementary_Information.pdf]

Cell Reports, Volume *44*

Supplemental information

A single-cell, long-read, isoform-resolved case-control study of FTD reveals cell-type-specific and broad splicing dysregulation in human brain

Natan Belchikov, Wen Hu, Li Fan, Anoushka Joglekar, Yi He, Careen Foord, Julien Jarroux, Justine Hsu, Shaun Pollard, Sadaf Amin, Andrey D. Prjibelski, Shiaoching Gong, Sai Zhang, Roberta Giannelli, Harro Seelaar, Alexandru I. Tomescu, M. Elizabeth Ross, Alissa Nana Li, Lea T. Grinberg, Salvatore Spina, Bruce L. Miller, Johnathan Cooper-Knock, Michael P. Snyder, William W. Seeley, Priyanka Rao-Ruiz, Sabine Spijker, August B. Smit, Claire D. Clelland, Li Gan, and Hagen U. Tilgner

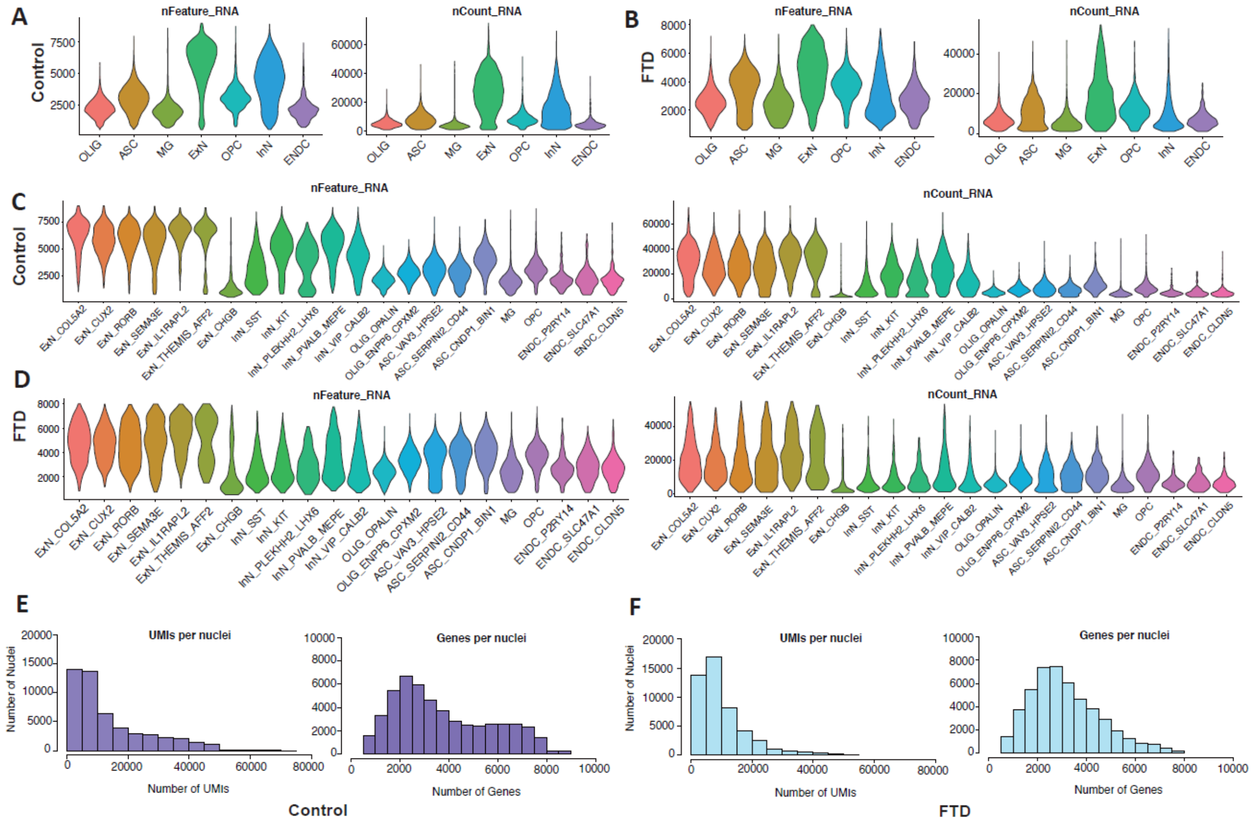

Figure S1: Gene and unique molecular identifier (UMI) distributions by cell type, subtype, and disease status.

(A–B) Violin plot of genes and UMIs detected per cell type in control and FTD groups. ASC, astrocytes; ExN, excitatory neurons; InN, inhibitory neurons; OLIG, oligodendrocytes; MG, microglia; OPC, oligodendrocyte precursor cells; ENDC, endothelial cells.
(C–D) Violin plot of genes and UMIs per cell subtype in control and FTD groups.
(E–F) Distribution of detected genes and UMIs per nucleus in control and FTD groups.

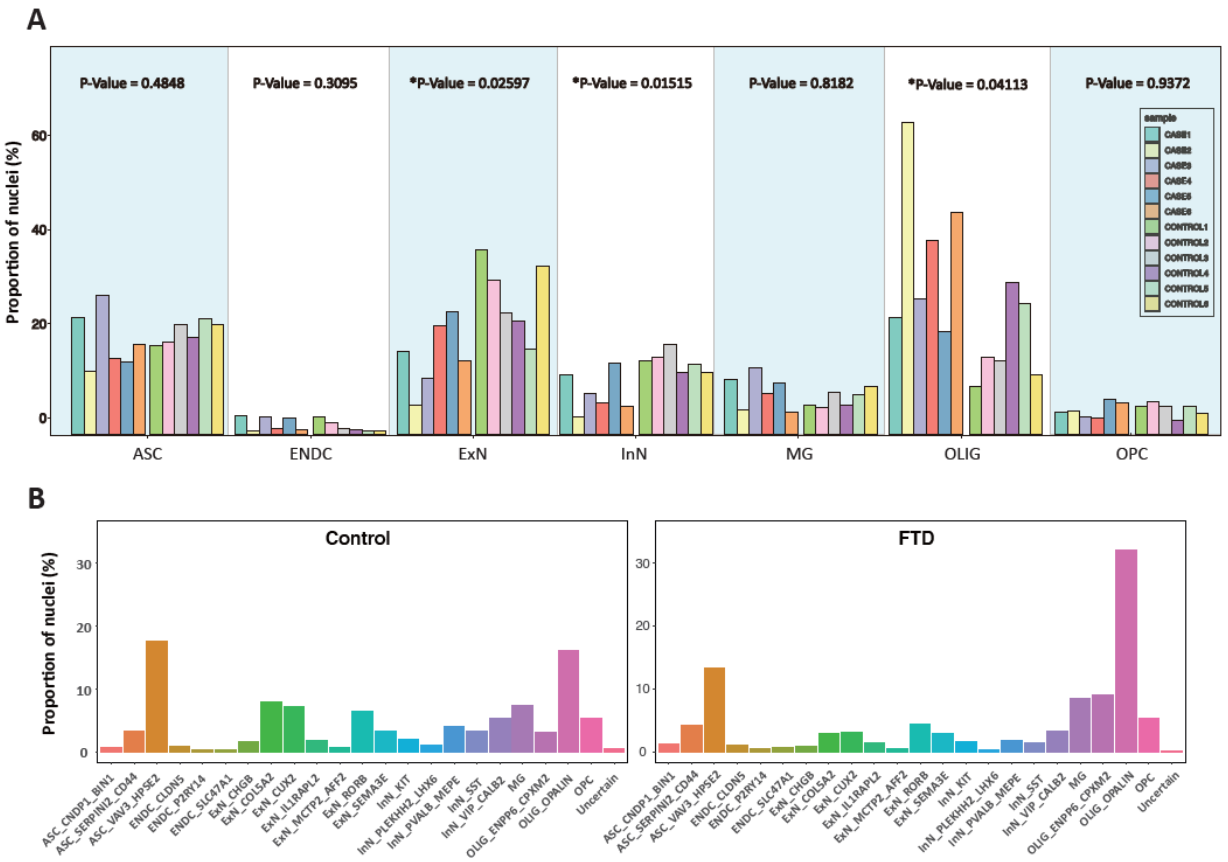

Figure S2: Variation in cellular composition by sample and disease status.

(A) Proportion of nuclei per cell type per sample (two-sided Wilcoxon rank sum test was performed between 6 controls and 6 FTD samples for each cell type, *p* value per cell type was indicated on top).
(B) Proportion of nuclei per subtype per condition.

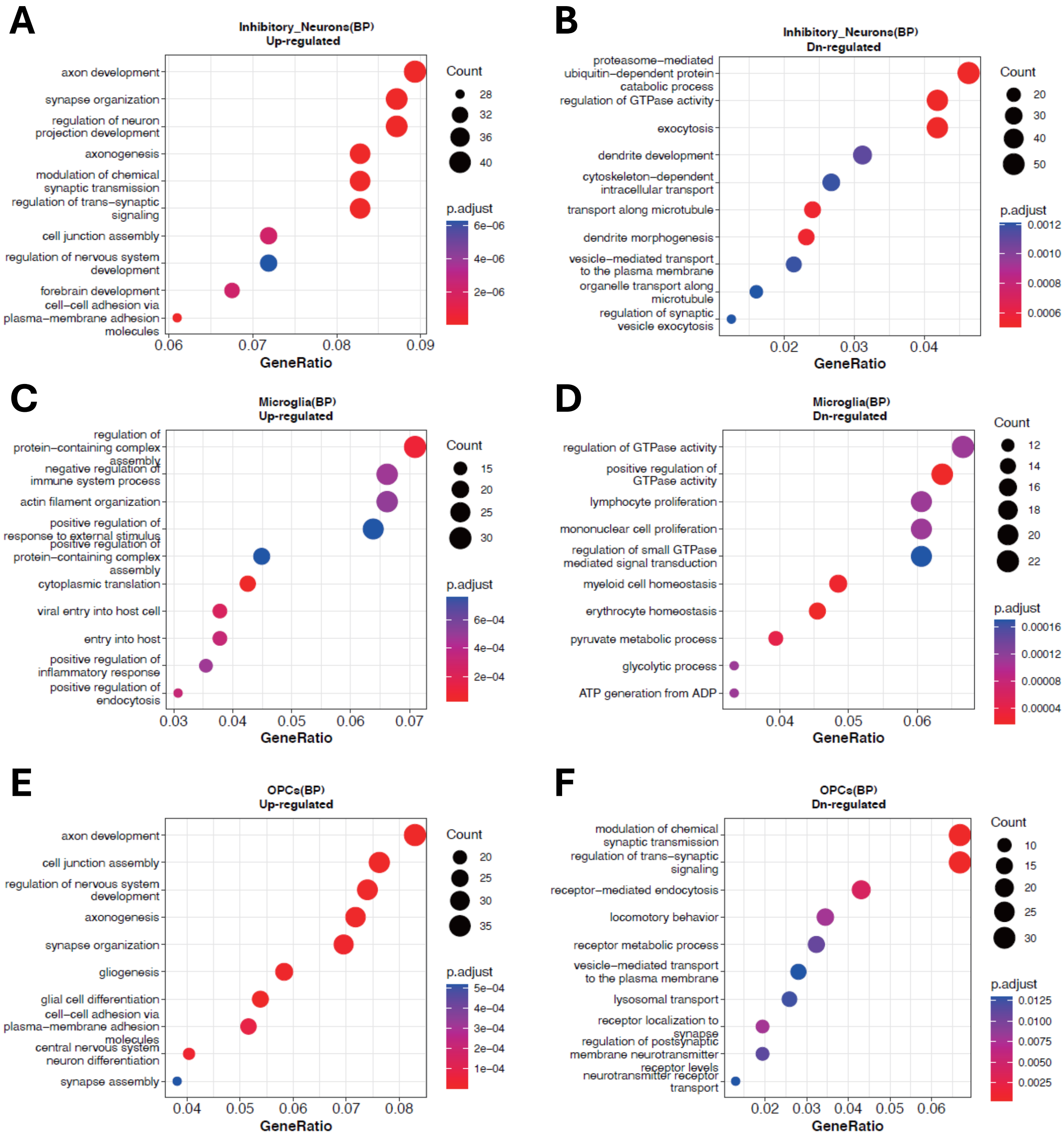

Figure S3: Gene Ontology (GO) analysis for inhibitory neurons, microglia, and OPCs.
GO terms enriched in (A) upregulated and (B) downregulated inhibitory neurons, (C) upregulated and (D) downregulated microglia, and (E) upregulated and (F) downregulated OPCs.

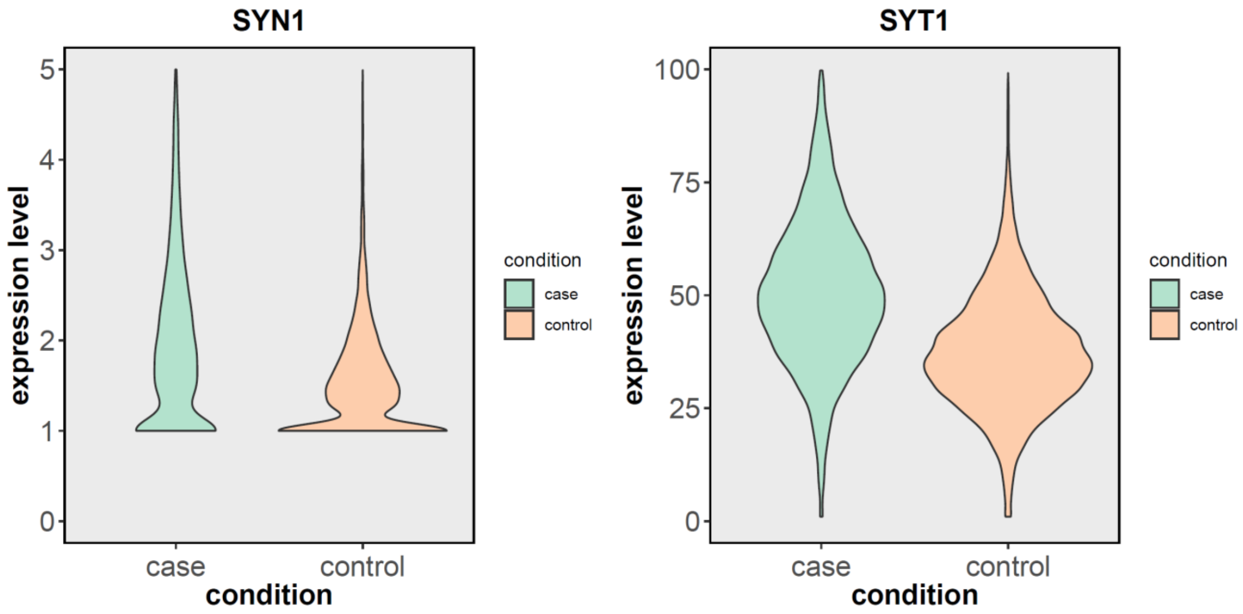

Figure S4: Two differentially expressed synaptic genes.
Gene expression levels of *SYN1* (left) and *SYT1* (right) for case and control samples.

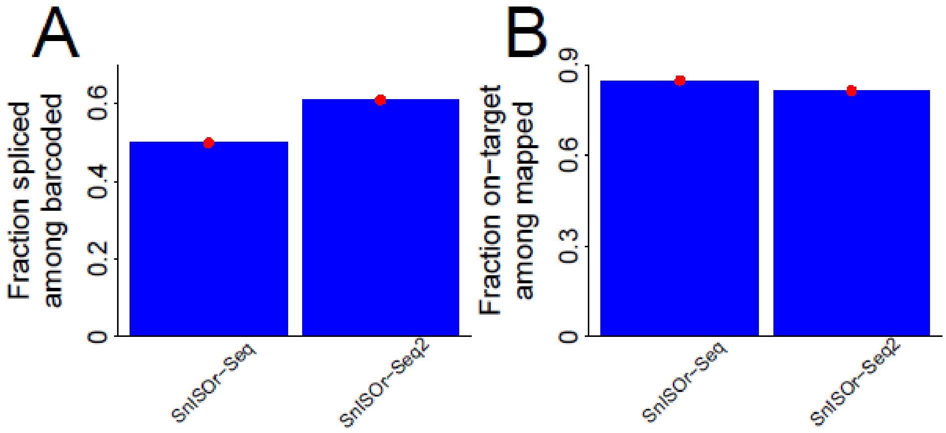

Figure S5: Comparison of proportions of spliced and on-target molecules by technology.

(A) Fraction of reads that were spliced among all barcoded reads. Calculated using 2 samples for SnISOr-Seq from a prior study and all 12 samples from the present study. Error bars indicate 95% confidence intervals.

(B) Fraction of reads that were on-target (overlapped a targeted gene) among all mapped reads. Calculated using 2 samples for SnISOr-Seq from a prior study and all 12 samples from the present study. Error bars indicate 95% confidence intervals.

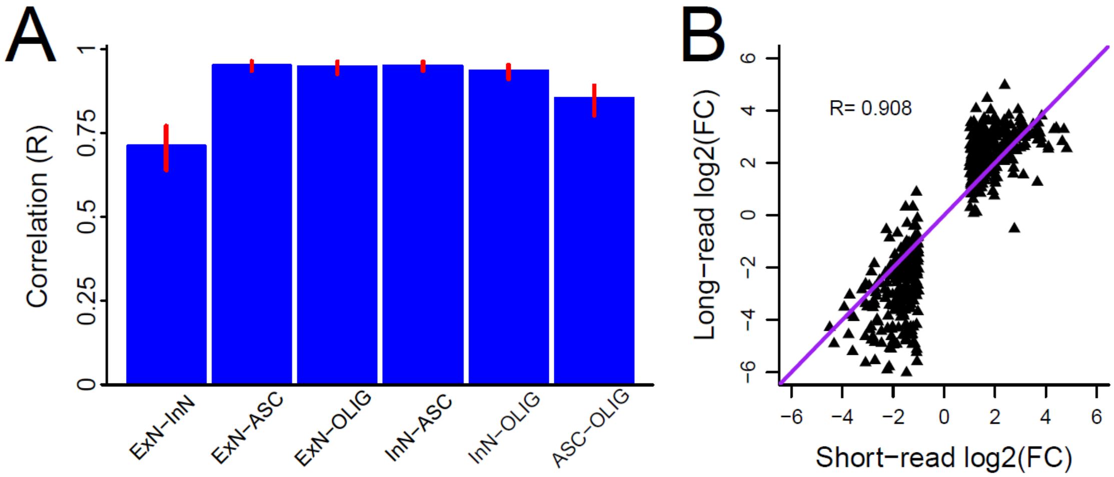

Figure S6: Cross-dataset and cross-platform validation of long-read sequencing approach.

(A) Bar-plot of correlations of $\Delta\Psi$ values for pairs of cell types between control samples and our previously published data. Error bars indicate 95% confidence intervals.

(B) Dot-plot of $\log_2$FC of gene expression between excitatory neurons and astrocytes as calculated in short-read data (*x*-axis) and long-read data (*y*-axis). Only genes with short-read $|\log_2\text{FC}| > 1$ are shown.

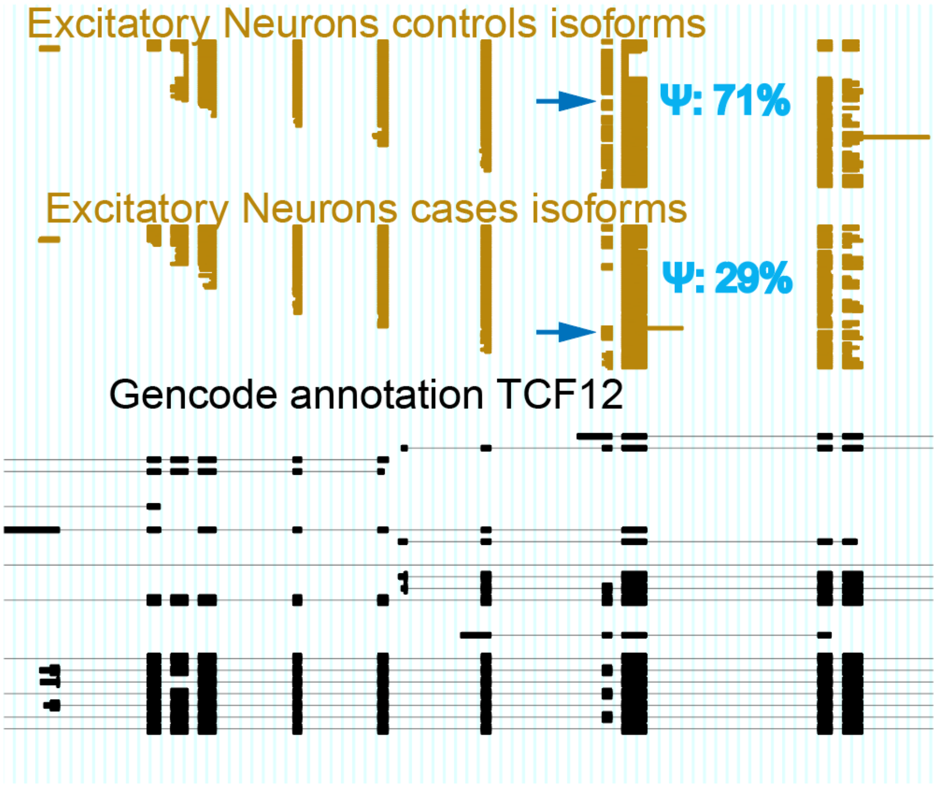

Figure S7: *TCF12* splicing patterns in excitatory neurons around a dysregulated exon. Excitatory neuron (tracks in light brown) for *TCF12*, showing similar behavior to that seen in inhibitory neurons (Figure 3C). Each line corresponds to one molecule. Reads from all case samples are grouped together; reads from all control samples are grouped together. Only informative reads for the highlighted exon are shown. Bottom (black) track: GENCODE annotation (v34) for *TCF12*.

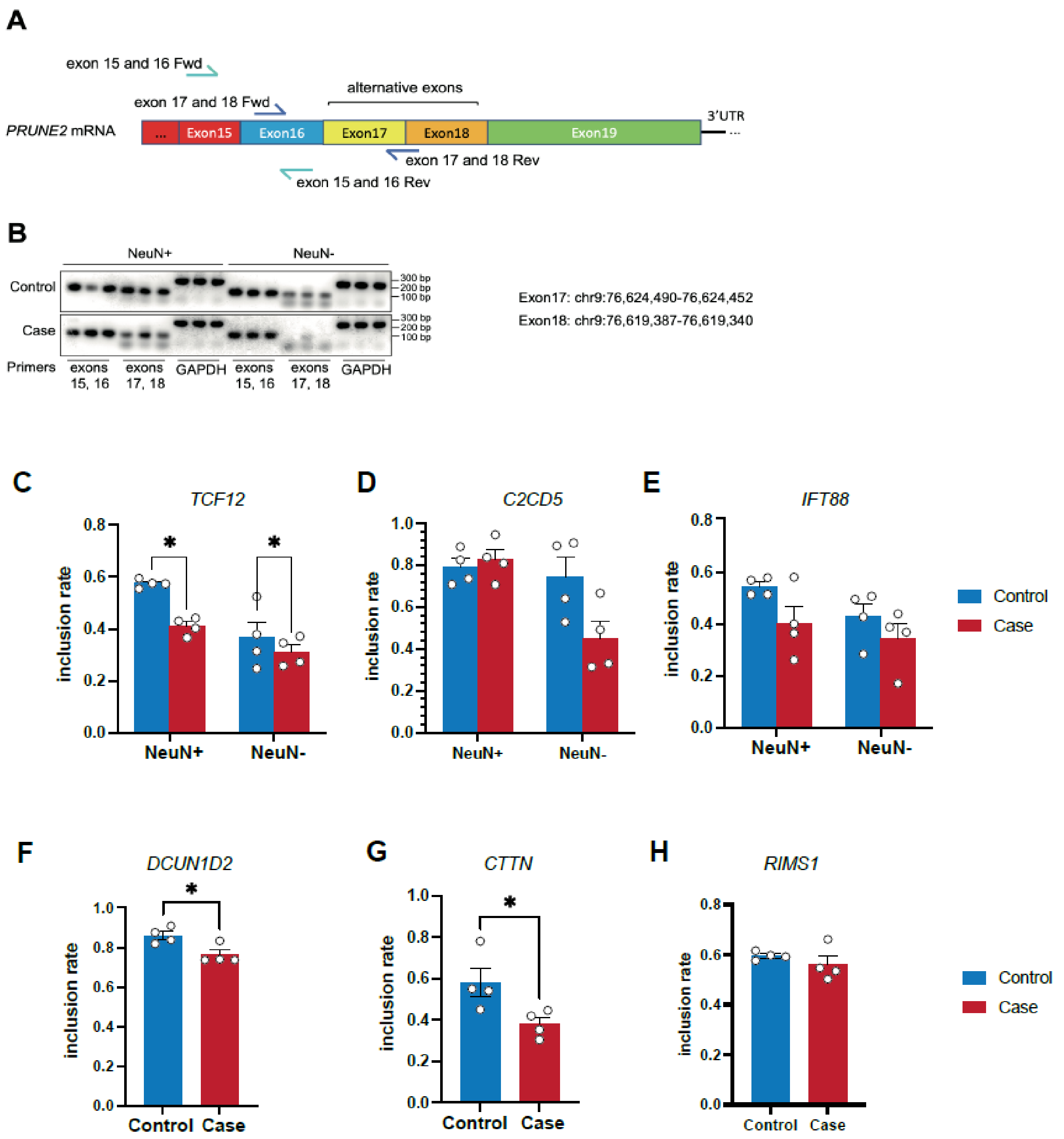

I

	Neurons		Non-neurons	
	Control Ψ	Case Ψ	Control Ψ	Case Ψ
TCF12	79%	28%	31%	16%
C2CD5	90%	89%	36%	14%
IFT88	37%	17%	29%	21%

	Control Ψ	Case Ψ
DCUN1D2	52%	43%
CTTN	41%	26%
RIMS1	36%	35%

Figure S8: Validation of select sequencing-based results with semi-quantitative reverse-transcription PCR (RT-sqPCR).

(A) Primer design and (B) RT-sqPCR DNA electrophoresis result of NeuN— and $NeuN^+$ nuclei populations for exons 17 and 18 of *PRUNE2*. *GAPDH* was the internal control for normalization for each condition.

(C–E) Validation of alternative-exon inclusion rate at the cell-type-specific level for three genes: *TCF12*, *C2CD5*, and *IFT88*. $n = 4$ for control, $n = 4$ for case. Statistical significance was calculated by two-way ANOVA, followed by Tukey test for multiple comparisons. * indicates $p < 0.05$. Error bars indicate SEM.

(F–H) Validation of alternative-exon inclusion rate at the bulk level for three genes: *DCUN1D2*, *CTTN*, and *RIMS1*. $n = 4$ for control, $n = 4$ for case. Statistical significance was calculated by unpaired t test.
* indicates $p < 0.05$. Error bars indicate SEM.

(I) Table of control Ψ and case Ψ values from sequencing of the same samples, in neurons (excitatory and inhibitory) and non-neurons (astrocytes, oligodendrocytes, and microglia) for *TCF12*, *C2CD5*, and *IFT88*; and in pseudobulk for *DCUN1D2*, *CTTN*, and *RIMS1*. Green indicates FDR < 0.05 and orange indicates FDR < 0.06 with Fisher's exact test and the Benjamini–Yekutieli correction for multiple comparisons. Sequencing data were used from only the 4 control and 4 FTD samples that were used in the RT-sqPCR experiments.

A

***SLC25A26*, chr3:66236544-66236700**

	inclusion in astrocytes	exclusion in astrocytes
**FTD cases**	91	41
**controls**	91	151

ΔΨ: 31%

	inclusion in pseudobulk	exclusion in pseudobulk
**FTD cases**	620	369
**controls**	1159	966

ΔΨ: 8%

B

***CEP97*, chr3:101728836-101728937**

	inclusion in oligodendrocytes	exclusion in oligodendrocytes
**FTD cases**	70	5
**controls**	28	15

ΔΨ: 28%

	inclusion in pseudobulk	exclusion in pseudobulk
**FTD cases**	119	14
**controls**	111	23

ΔΨ: 7%

Figure S9: Examples of masking of cell-type-specific dysregulation events in pseudobulk data.
(A) 2×2 contingency tables for astrocytes (top) and pseudobulk (bottom) for an alternative exon in the *SLC25A26* gene.
(B) 2×2 contingency tables for oligodendrocytes (top) and pseudobulk (bottom) for an alternative exon in the *CEP97* gene.

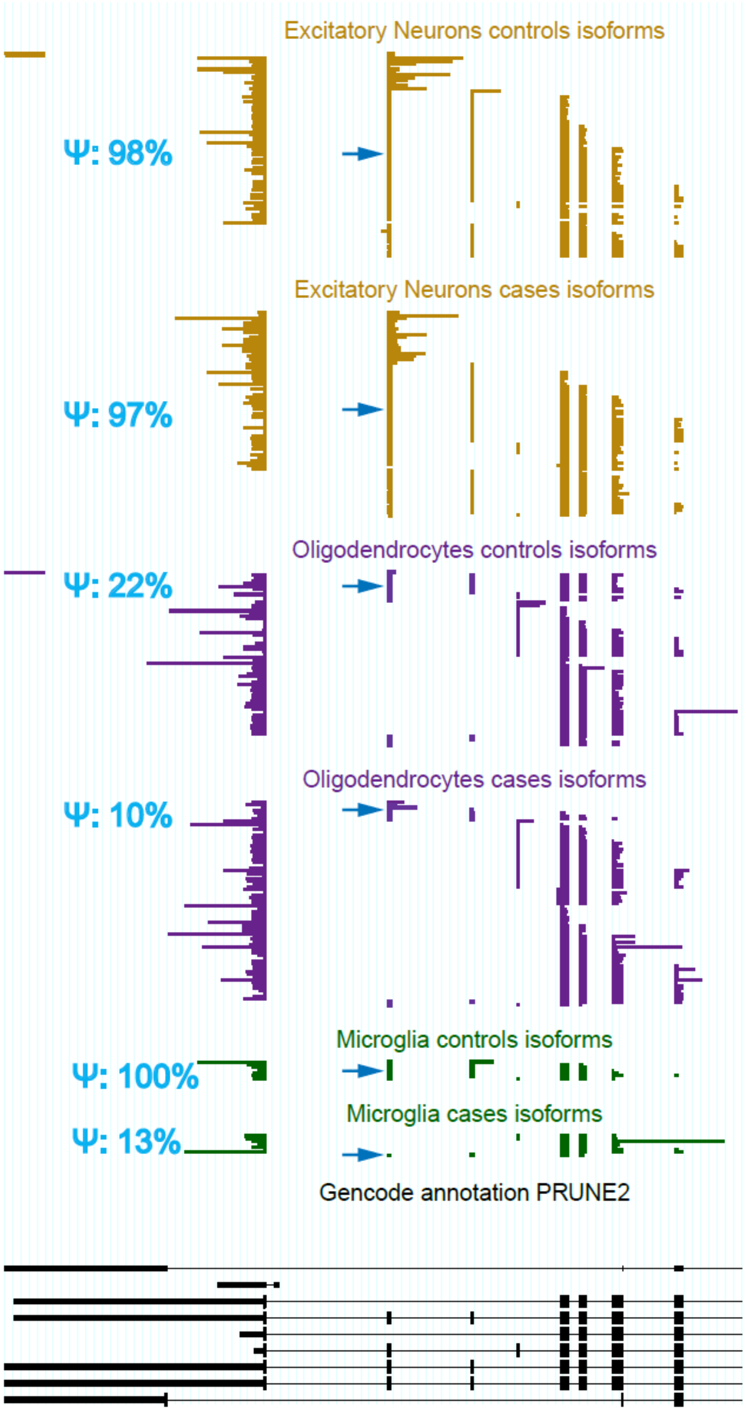

Figure S10: *PRUNE2* splicing patterns in excitatory neurons, oligodendrocytes, and microglia around a dysregulated exon.

Excitatory neuron (top tracks in light brown), oligodendrocyte (middle tracks in purple), and microglia (bottom tracks in green) data for *PRUNE2*. Each line corresponds to one molecule. Reads from all case samples are grouped together; reads from all control samples are grouped together. Only informative reads for the highlighted exon (exon 18, chr9:76,619,387-76,619,340) are shown. The exon immediately to the right (exon 17, chr9:76,624,490-76,624,452) behaves largely in a coordinated manner, as in inhibitory neurons and astrocytes (Fig 4E). Bottom (black) track: GENCODE annotation (v34) for *PRUNE2*.

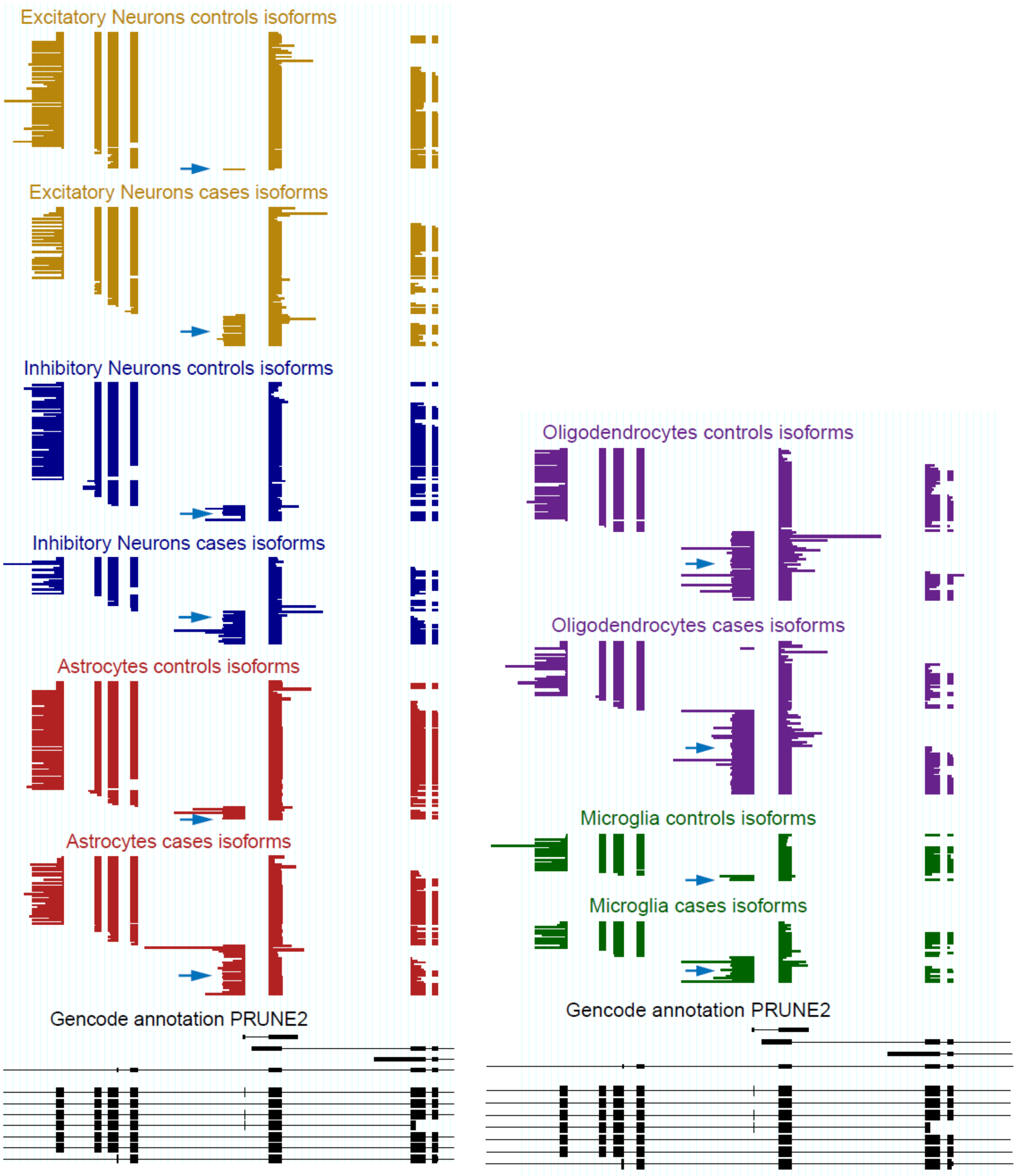

Figure S11: *PRUNE2* splicing patterns around a previously identified cryptic exon. Excitatory neuron (top left tracks in light brown), inhibitory neuron (middle left tracks in blue), astrocyte (bottom left tracks in red), oligodendrocyte (top right tracks in purple) and microglia (bottom right tracks in green) data for *PRUNE2* cryptic exon (within the intron chr9:76638288-76644739). Each line corresponds to one molecule. Reads from all case samples are grouped together; reads from all control samples are grouped together. Only reads overlapping the highlighted exon's 5' (rightward) splice site are shown. Bottom (black) track: GENCODE annotation (v34) for *PRUNE2*.

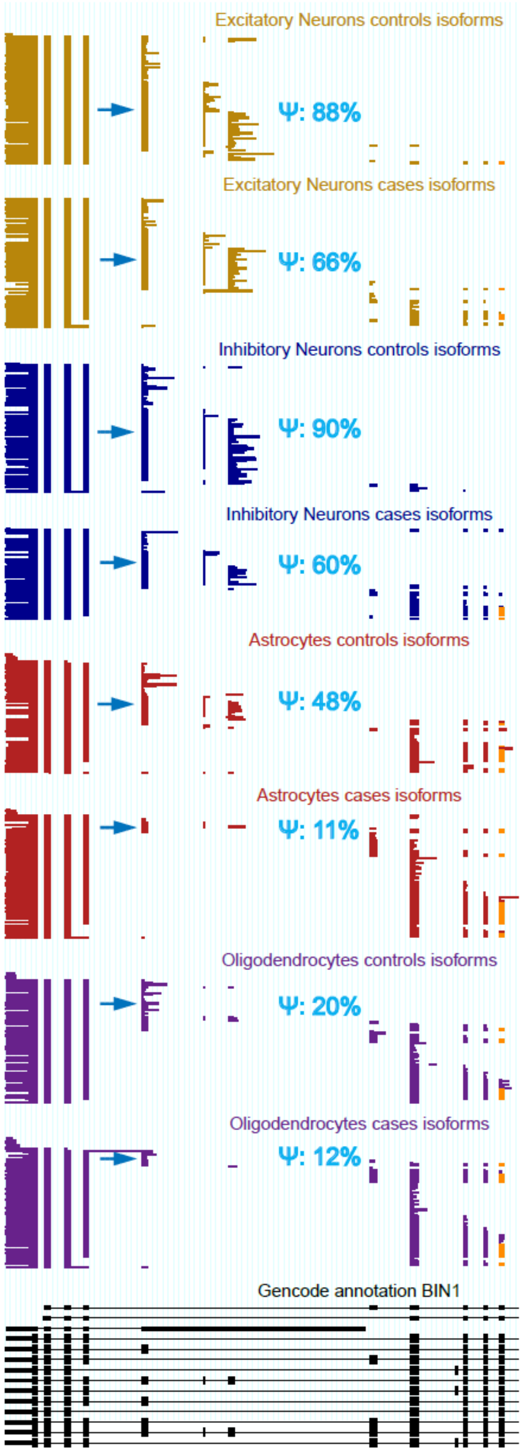

Figure S12: *BIN1* splicing patterns around a dysregulated exon.
Excitatory neuron (top tracks in light brown), inhibitory neuron (top-middle tracks in blue), astrocyte (bottom-middle tracks in red), and oligodendrocyte (bottom tracks in purple) data for *BIN1*. Each line corresponds to one molecule. Reads from all case samples are grouped together; reads from all control samples are grouped together. Only informative reads for the highlighted exon are shown. Bottom (black) track: GENCODE annotation (v34) for *BIN1*.

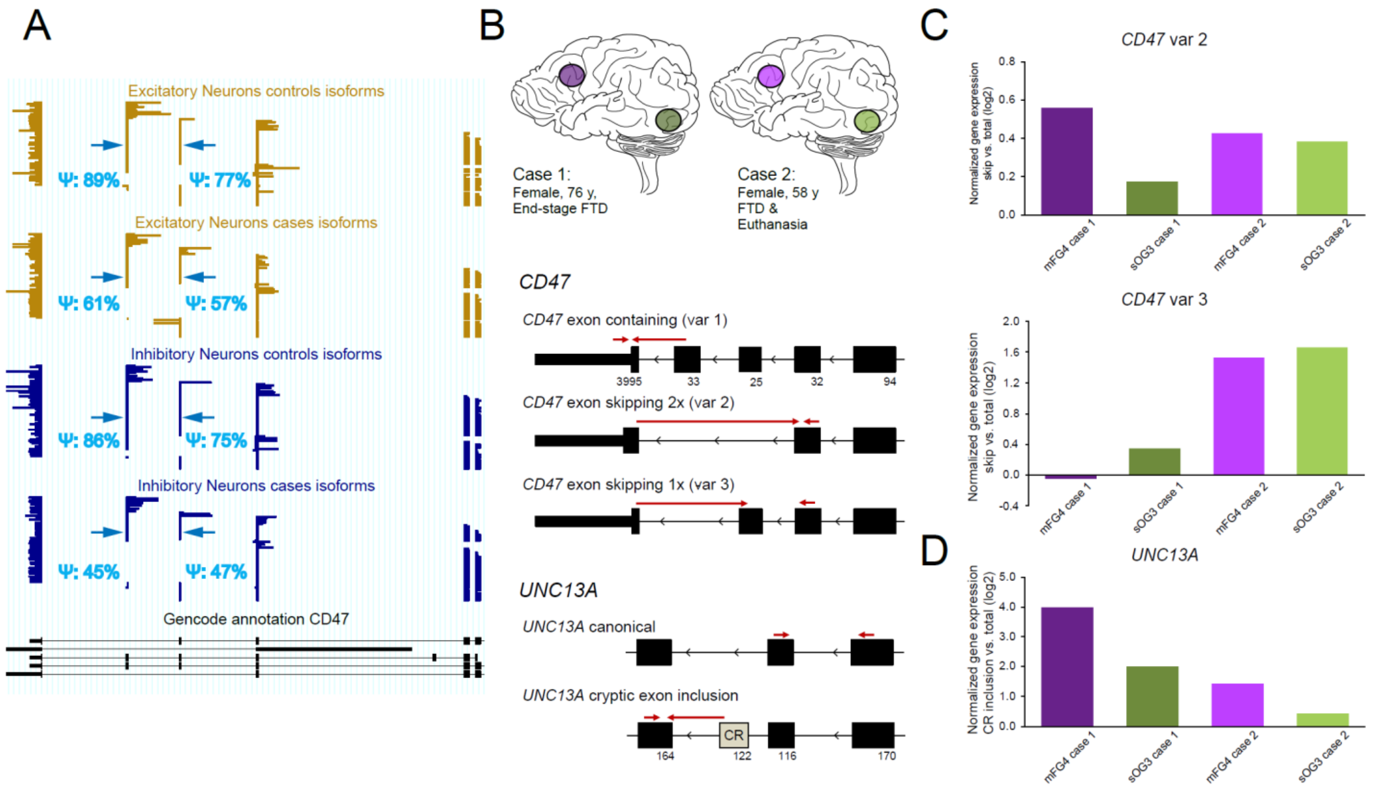

Figure S13: Validation of expression of *CD47* alternative exons and measurement of *UNC13A* cryptic-exon expression in differentially affected brain areas from independent GRN-FTD samples.

(A) Excitatory neuron (top tracks in light brown) and inhibitory neuron (blue) sequencing data for *CD47*. Each line corresponds to one molecule. Reads from all case samples are grouped together; reads from all control samples are grouped together. Only informative reads for the highlighted exons are shown. Bottom (black) track: GENCODE annotation (v34) for *CD47*.

(B) Experimental set-up (top) for quantitative real-time reverse-transcription PCR (RT-qPCR) analysis of splicing events for left middle frontal gyrus (purple) versus left superior occipital gyrus (green) in two GRN-FTD patients. Overview of *CD47* (middle) and *UNC13A* (bottom) alternatively spliced exons and splicing-specific qPCR primer design. Numbers below exons indicate their size in nucleotides.

(C) Validation of expression of *CD47* (top, middle) alternative-exon usage by RT-qPCR in frontal and occipital brain areas. Quantitative analysis of the FTD-specific exclusion rate of exons 9 and 10 (variant 2; NM_198793) or exclusion rate of exon 10 (variant 3; NM_001382306) was performed by normalizing with the expression level of transcripts that include exons 9 and 10 (variant 1; NM_001777). The variant 2 transcript—marked by the absence of both exons 9 and 10—shows brain-region specific splicing in the frontal versus occipital area in GRN-FTD case 1 (log_2FC case 1: 0.39). In case 2, this splicing is present, but there was no overt difference (log_2FC case 2: 0.05). On the other hand, the variant 3 transcript—marked by the absence of exon 10 only—also shows brain-region specific splicing in the frontal versus occipital area, but in an opposite manner, namely less occurrence in the frontal than the occipital region (log_2FC case 1: −0.38; log_2FC case 2: −0.14).

(D) Expression (measured with RT-qPCR) of the *UNC13A* alternative exon in frontal and occipital brain areas. Quantitative analysis of the inclusion rate of the cryptic exon was performed by normalizing with the expression level of the canonical transcript devoid of this exon. Brain-region specific splicing was observed in the frontal versus occipital area in both patients (log_2FC case 1: 1.98; log_2FC case 2: 1.01). Disease progression appeared to correlate with the rate of splicing, as a higher proportion of the FTD-specific splicing events was detected in GRN-FTD case 1, independent of brain region (C, D).

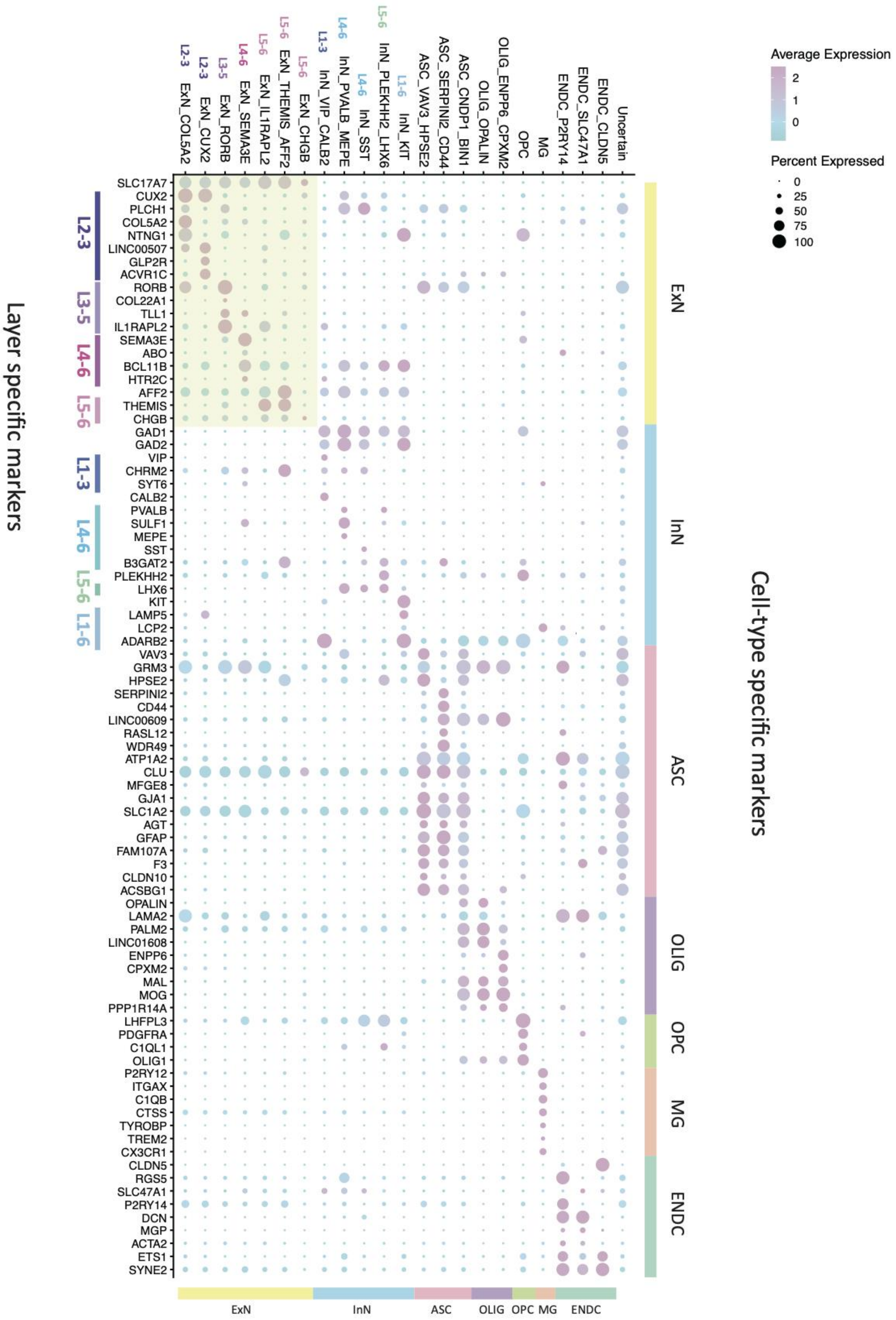

Figure S14: Markers for neuronal subtypes and layers.

Cell-type-specific markers and layer-specific markers used for defining cell subtypes and corresponding layers (excitatory and inhibitory neurons only).

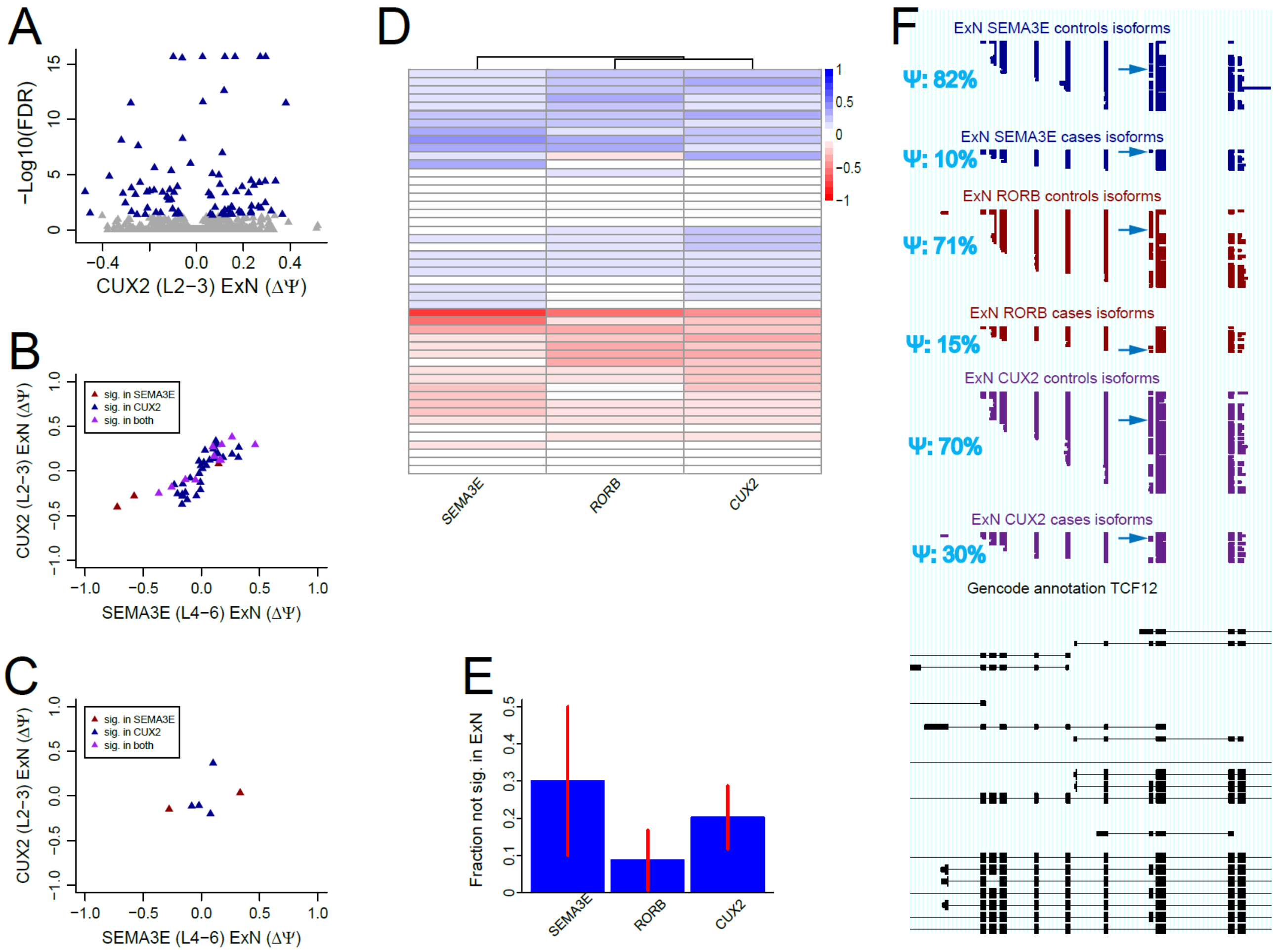

Figure S15: Splicing dysregulation in distinct cortical layers.

(A) Volcano plot of L2–3 (*CUX2*-marked) excitatory neuron exon inclusion of cases vs. controls. *x*-axis: ΔΨ for each exon; *y*-axis: $-\log_{10}$(adjusted *p* value).

(B) ΔΨ values for cases vs. controls for L4–6 (*SEMA3E*-marked) excitatory neurons (*x*-axis) and L2–3 (*CUX2*-marked) excitatory neurons (*y*-axis) for exons that showed significance in all excitatory neurons.

(C) Same plot as (B) for exons that did not show significance in all excitatory neurons.

(D) Heatmap of ΔΨ for exons (each exon is one row) in three subtypes of excitatory neurons associated with distinct cortical layers.

(E) Fraction of exons that are significant in an excitatory subtype but not in excitatory neurons overall. Error bars indicate 95% confidence intervals.

(F) Excitatory neuron subtype data, following the same conventions as in previous figures, for *TCF12*.

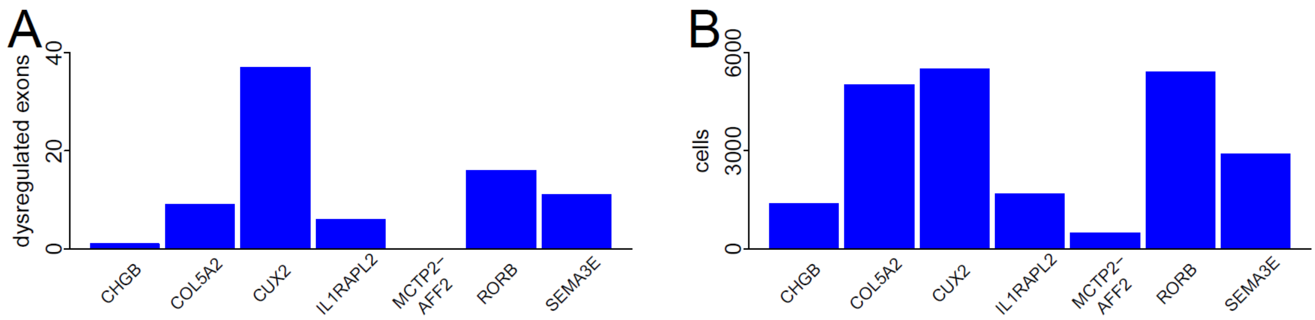

Figure S16: Analysis across all subtypes of excitatory neurons.
(A) Number of dysregulated exons per excitatory-neuron subtype.
(B) Number of total nuclei per excitatory-neuron subtype.

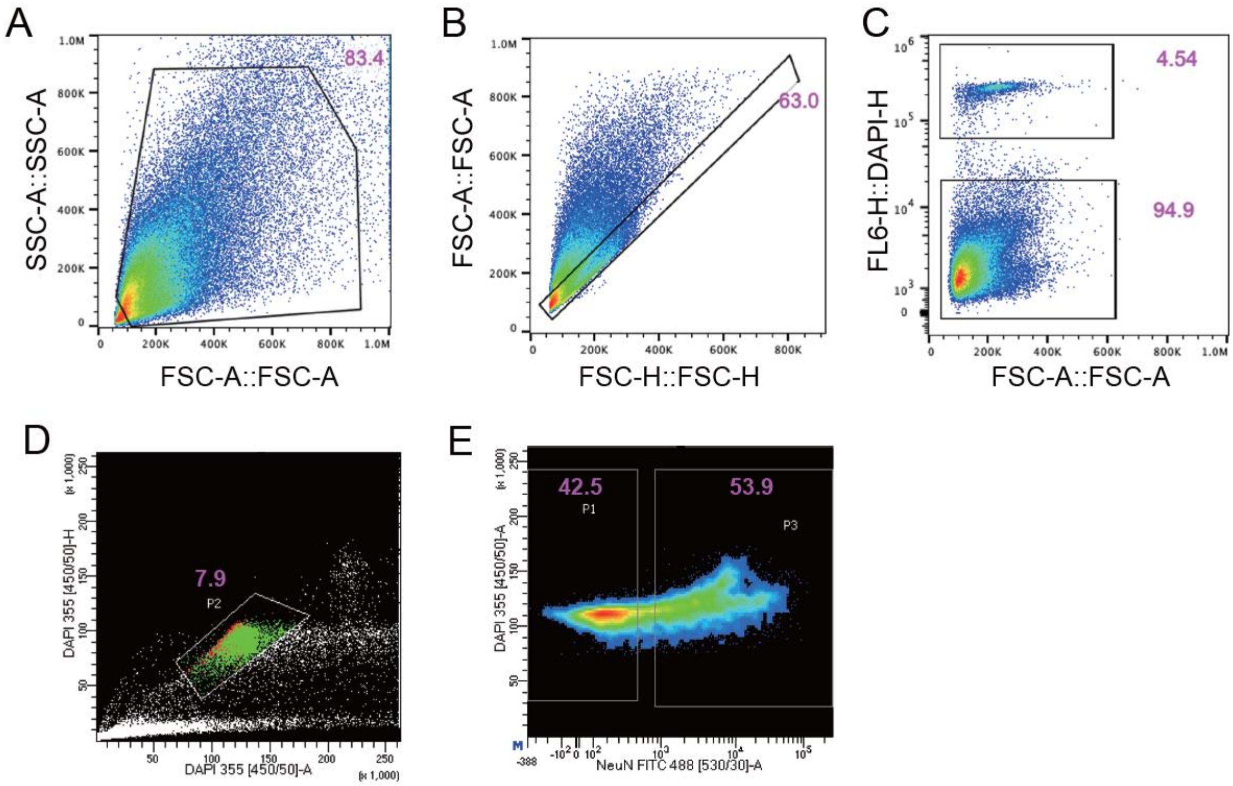

Figure S17: Flow cytometry plots.

Representative image of FACS gating strategy used.

(A) Most events were included in the FSC/SSC gate.

(B) Singlets were identified from the FSC-H/FSC-A gate.

(C) Lastly, a distinct DAPI+ population was sorted.

(D) For *PRUNE2* RT-sqPCR validation, a $DAPI^{+}$ population was gated using DAPI-A/DAPI-H.

(E) $NeuN^{+}$ and $NeuN^{-}$ populations were separated and sorted from NeuN-488-A gate.

Table S1: Donor information for sequenced samples.

Table of characteristics pertaining to the 12 donors whose brain tissues were sequenced in this study.

Sample ID	Brain bank ID	Sex	Age	Clinical	Primary Neuropath Dx	PMI (hrs)	AD Thal Phase	AD/PD Braak Stage	AD CERAD NP Score	ADNC level	LBD Stage
FTD_1	P2503	M	64	bvFTD	FTLD-TDP-A	7.2	0	2/0	0	0	0
FTD_2	P2805	F	56	bvFTD	FTLD-TDP-A	7.6	2	1/0	0	1	0
FTD_3	P2843	F	64	CBS	FTLD-TDP-A	10.5	2	1/0	0	1	0
FTD_4	P2920	F	63	bvFTD	FTLD-TDP-A	10.4	4	1/0	2	1	0
FTD_5	P2943	F	74	nfvPPA	FTLD-TDP-A	2.2	2	2/0	1	1	0
FTD_6	P3040	F	84	bvFTD	FTLD-TDP-A	10.7	2	3/0	1	1	0
Ctrl_1	P2921	F	82	Control	None	9.6	2	1/0	0	1	0
Ctrl_2	P2937	F	60	Control	None	20.5	3	0/0	0	1	0
Ctrl_3	P2942	F	67	Control	None	19.4	0	0/0	0	0	0
Ctrl_4	P2947	M	95	Control	Non	9.6	3	4/0	3	2	0
Ctrl_5	P3006	M	91	Control	None	5.6	2	4/0	1	1	0
Ctrl_6	P3041	F	78	Control	None	11.6	1	4/0	0	1	0

Table S2: Dysregulated exons by cell type.

List of exons that were found to be significant in each of the five major cell types, providing their coordinates, strand and gene information, and values for ΔΨ, FDR, and log-odds ratio. An exon was considered to be significant when FDR ≤ 0.05, |ΔΨ| ≥ 20%, and individual-sample requirements were met (see main text). An asterisk indicates a high-confidence exon, meaning the ΔΨ calculated using a subset of 8 samples that were better matched in age than the full set was at least 50% of the full-set ΔΨ.

**Excitatory neurons**					
**Exon**	**gene**	**ENSEMBL ID**	**ΔΨ**	**FDR**	**log-odds ratio**
chr15_57252421_57252492_+*	TCF12	ENSG00000140262.18	-0.417398	2.48E-07	-2.56551
chr15_73274692_73274724_+*	NEO1	ENSG00000067141.17	-0.355462	0.03321965	-2.333508
chr2_159764626_159764674_+*	MARCHF7	ENSG00000136536.15	-0.352843	0.01026826	-2.813231
chr3_138619711_138619770_+*	FAIM	ENSG00000158234.12	-0.326399	0.03233958	-1.991924
chr12_101684382_101684424_+*	MYBPC1	ENSG00000196091.15	-0.314346	3.97E-06	-1.927785
chr9_36209267_36209320_+*	CLTA	ENSG00000122705.17	-0.296789	1.82E-39	-2.012196
chr8_86542046_86542074_+*	CPNE3	ENSG00000085719.13	-0.291808	5.17E-17	-1.788798
chr3_197068502_197068537_-*	DLG1	ENSG00000075711.21	-0.277447	8.95E-07	-1.657926
chr3_108049619_108049651_-*	CD47	ENSG00000196776.16	-0.274347	7.22E-20	-2.317796
chr12_88054340_88054413_-*	CEP290	ENSG00000198707.16	-0.268291	3.31E-14	-1.589104
chr7_94587774_94587812_-*	SGCE	ENSG00000127990.19	-0.256547	3.49E-26	-1.943904
chr20_53975389_53975430_-*	BCAS1	ENSG00000064787.13	-0.233808	0.03332661	-1.375891
chr3_142186194_142186267_-*	GK5	ENSG00000175066.16	-0.230972	0.005572107	-1.68859
chr4_76147479_76147640_-*	NUP54	ENSG00000138750.15	-0.228281	0.009108592	-1.38665
chr20_8802056_8802173_+*	PLCB1	ENSG00000182621.18	-0.225749	0.008387834	-1.542064
chr2_127052255_127052362_-*	BIN1	ENSG00000136717.15	-0.22266	2.68E-10	-1.919086
chr8_38823990_38824025_+*	TACC1	ENSG00000147526.20	-0.220926	3.97E-07	-1.339349
chr9_108983417_108983459_-*	CTNNAL1	ENSG00000119326.15	-0.212382	3.99E-07	-1.244585
chr7_35181415_35181502_-*	DPY19L2P1	ENSG00000189212.12	-0.21207	0.001013816	-1.251045
chr3_108050578_108050602_-*	CD47	ENSG00000196776.16	-0.208816	5.67E-09	-1.397773
chr11_70365373_70365435_+*	PPFIA1	ENSG00000131626.18	-0.201807	0.000792779	-1.298224
chr21_26000015_26000182_-*	APP	ENSG00000142192.21	0.203669	9.57E-05	1.262561
chr3_57864807_57864857_+*	SLMAP	ENSG00000163681.16	0.203921	0.04735409	1.205709
chr1_20780345_20780540_-*	HP1BP3	ENSG00000127483.19	0.204543	1.05E-147	1.259065
chr10_122428276_122428316_+*	PLEKHA1	ENSG00000107679.14	0.204948	1.23E-06	1.340166
chr15_33816862_33816958_+	RYR3	ENSG00000198838.13	0.207072	0.001862655	1.215213
chr11_115209574_115209657_-*	CADM1	ENSG00000182985.17	0.20742	1.03E-10	1.298008
chr1_35506612_35507135_-*	KIAA0319L	ENSG00000142687.18	0.208728	0.00337065	1.228159
chr3_38234565_38234760_+*	OXSR1	ENSG00000172939.9	0.208905	0.002744771	2.110143
chr2_152679056_152679109_-*	PRPF40A	ENSG00000196504.18	0.221176	1.06E-06	1.715168
chr7_111784097_111784123_-*	DOCK4	ENSG00000128512.23	0.221382	0.01479601	1.299082
chr13_21381901_21381945_-*	ZDHHC20	ENSG00000180776.15	0.221807	8.87E-05	1.604397
chr2_95919733_95919805_-*	ANKRD36C	ENSG00000174501.14	0.22742	9.69E-13	1.770505
chr4_48857213_48857365_+*	OCIAD1	ENSG00000109180.14	0.24371	4.46E-36	1.499497
chr12_50671186_50671398_+*	DIP2B	ENSG00000066084.13	0.244279	0.00042976	1.439686
chr11_105971914_105972028_+*	GRIA4	ENSG00000152578.13	0.250641	0.000605315	2.026547
chr9_105356212_105356381_+*	SLC44A1	ENSG00000070214.16	0.253487	0.02418801	1.587313

chrX_38287861_38288041_-*	RPGR	ENSG00000156313.15	0.254049	0.02272969	1.565854
chr2_241324216_241324241_+*	SEPTIN2	ENSG00000168385.18	0.255716	1.72E-09	1.516442
chr14_21022864_21022905_-*	NDRG2	ENSG00000165795.23	0.261842	0.001013896	1.549601
chr1_156658127_156658271_+*	BCAN	ENSG00000132692.19	0.278751	0.000380636	1.682727
chr5_96740745_96740783_+*	CAST	ENSG00000153113.23	0.316104	1.39E-28	3.734975
chr16_10414574_10414612_+*	ATF7IP2	ENSG00000166669.13	0.356759	0.01296373	2.84666
chr10_95395001_95395075_-*	SORBS1	ENSG00000095637.22	0.364089	3.88E-18	2.82638
chr2_165179552_165179691_-*	SCN3A	ENSG00000153253.18	0.38473	0.01608441	3.242458
chr9_4626426_4626555_-*	SPATA6L	ENSG00000106686.16	0.407407	0.004976769	2.646729
chr17_50975863_50975901_-*	SPAG9	ENSG00000008294.21	0.633333	0.006898181	4.321928

**Inhibitory neurons**					
**exon**	**gene**	**ENSEMBL ID**	**ΔΨ**	**FDR**	**log-odds ratio**
chr6_128003197_128003232_-*	PTPRK	ENSG00000152894.14	-0.52963	0.003587885	-3.93546
chr7_94587774_94587812_-*	SGCE	ENSG00000127990.19	-0.485704	4.95E-18	-3.201019
chr15_57252421_57252492_+*	TCF12	ENSG00000140262.18	-0.450891	0.003289984	-2.808015
chr3_108049619_108049651_-*	CD47	ENSG00000196776.16	-0.409085	9.53E-08	-2.876194
chr9_36209267_36209320_+*	CLTA	ENSG00000122705.17	-0.341026	9.91E-13	-2.366322
chr2_127052255_127052362_-*	BIN1	ENSG00000136717.15	-0.297436	0.000399238	-2.544321
chr2_232700585_232700654_+*	GIGYF2	ENSG00000204120.15	-0.294785	0.04770965	-1.88835
chr5_176396479_176396532_-*	CLTB	ENSG00000175416.15	-0.291913	1.21E-05	-2.612829
chr7_152224015_152224179_-*	KMT2C	ENSG00000055609.18	-0.287289	0.00700474	-1.807159
chr3_108050578_108050602_-*	CD47	ENSG00000196776.16	-0.284566	0.00381617	-1.789187
chr20_37238402_37238449_+*	RPN2	ENSG00000118705.17	-0.268286	0.00073082	-1.627207
chr9_92875015_92875059_-*	ZNF484	ENSG00000127081.14	-0.264442	0.03944225	-1.563388
chr3_121836645_121836794_+*	EAF2	ENSG00000145088.9	-0.229912	0.003169657	-1.996296
chr16_7693315_7693367_+*	RBFOX1	ENSG00000078328.21	-0.201749	0.001309563	-1.241454
chr4_48857213_48857365_+*	OCIAD1	ENSG00000109180.14	0.221833	1.36E-06	1.532774
chr2_97207934_97208006_+*	ANKRD36	ENSG00000135976.20	0.223023	0.004827981	1.356405
chr11_115198406_115198438_-*	CADM1	ENSG00000182985.17	0.240683	0.002737447	1.492442
chr5_138018955_138019146_-*	FAM13B	ENSG00000031003.10	0.242938	0.007791489	3.4476
chr2_95916610_95916721_-*	ANKRD36C	ENSG00000174501.14	0.243342	0.000193665	1.559118
chr13_95602423_95602688_-*	DZIP1	ENSG00000134874.17	0.268256	5.26E-06	2.544693
chr7_122034095_122034115_+*	PTPRZ1	ENSG00000106278.12	0.273994	0.0421247	1.627234
chr5_96740745_96740783_+*	CAST	ENSG00000153113.23	0.276408	0.000239526	2.809242
chr2_171022872_171023129_-*	TLK1	ENSG00000198586.14	0.287829	0.000241614	1.74881
chr5_179963471_179963564_-*	RNF130	ENSG00000113269.14	0.311966	1.71E-05	2.932886
chr7_128814638_128814919_+*	CCDC136	ENSG00000128596.17	0.359275	0.000474137	2.8129
chr3_57864807_57864857_+*	SLMAP	ENSG00000163681.16	0.37451	0.04031179	2.560088
chr15_84679932_84679969_-*	SEC11A	ENSG00000140612.14	0.391799	0.004239675	3.726795
chr7_128812708_128812929_+*	CCDC136	ENSG00000128596.17	0.419622	1.42E-06	3.738272
chr10_95395001_95395075_-*	SORBS1	ENSG00000095637.22	0.561864	9.09E-11	3.807355

Astrocytes					
**exon**	**gene**	**ENSEMBL ID**	**ΔΨ**	**FDR**	**log-odds ratio**
chr6_128003197_128003232_-*	PTPRK	ENSG00000152894.14	-0.397104	0.02069893	-2.545863
chr2_127052255_127052362_-*	BIN1	ENSG00000136717.15	-0.370235	0.000217259	-2.870365
chr12_27655143_27655235_+*	PPFIBP1	ENSG00000110841.14	-0.331622	3.01E-07	-2.289089
chr1_37496707_37496736_-*	MEAF6	ENSG00000163875.15	-0.330508	0.04983431	-2.292782
chr15_71892965_71893177_-*	MYO9A	ENSG00000066933.16	-0.306563	0.01815422	-1.965784
chr4_138182297_138182393_-*	SLC7A11	ENSG00000151012.13	-0.299837	5.16E-05	-1.934734
chr4_20712788_20712922_+*	PACRGL	ENSG00000163138.19	-0.297682	0.04717513	-1.771687
chrX_96735758_96735790_+*	DIAPH2	ENSG00000147202.18	-0.296677	2.26E-12	-4.349834
chr3_155486147_155486206_-*	PLCH1	ENSG00000114805.17	-0.286341	0.009823729	-3.703357
chr12_79806166_79806333_-*	PPP1R12A	ENSG00000058272.19	-0.26	2.42E-19	-1.579395
chr9_121094888_121095018_+*	CNTRL	ENSG00000119397.16	-0.255002	2.03E-06	-1.594896
chr12_88054340_88054413_-*	CEP290	ENSG00000198707.16	-0.243559	0.000489949	-1.648339
chr1_91396293_91396405_-*	HFM1	ENSG00000162669.16	-0.239621	0.0301101	-1.815125
chr9_36209267_36209320_+*	CLTA	ENSG00000122705.17	-0.237404	1.31E-09	-1.617935
chr1_236243422_236243495_-*	ERO1B	ENSG00000086619.14	-0.224045	1.50E-05	-3.273018
chr2_127053422_127053445_-*	BIN1	ENSG00000136717.15	-0.221994	0.01001138	-4.647059
chr18_13038370_13038579_+*	CEP192	ENSG00000101639.18	-0.219216	6.90E-05	-1.29844
chr6_83096731_83096823_+*	DOP1A	ENSG00000083097.14	-0.219111	0.002821983	-1.563092
chr8_103910134_103910201_+*	RIMS2	ENSG00000176406.23	-0.210273	0.03333391	-1.629412
chr15_85658537_85658590_+*	AKAP13	ENSG00000170776.22	-0.203886	0.04884622	-1.21659
chr2_25387289_25387378_-*	DTNB	ENSG00000138101.18	0.209808	0.007729742	1.512871
chr2_97207811_97207839_+*	ANKRD36	ENSG00000135976.20	0.21287	0.03584709	1.26437
chr10_60059698_60060006_-*	ANK3	ENSG00000151150.22	0.218169	0.007259824	1.303745
chr2_97206063_97206135_+*	ANKRD36	ENSG00000135976.20	0.231563	0.003986543	1.41517
chr2_97205940_97205968_+*	ANKRD36	ENSG00000135976.20	0.232858	0.007882666	1.39889
chr5_96740745_96740783_+*	CAST	ENSG00000153113.23	0.235881	1.36E-05	1.69903
chr8_103910321_103910529_+*	RIMS2	ENSG00000176406.23	0.237149	0.008298083	1.722986
chr2_227328678_227328789_+*	MFF	ENSG00000168958.20	0.267377	0.000776838	1.600644
chr2_97207934_97208006_+*	ANKRD36	ENSG00000135976.20	0.27186	0.000209632	1.675292
chr1_36287477_36287871_+*	THRAP3	ENSG00000054118.15	0.29845	0.001732093	3.790077
chr3_66236544_66236700_+*	SLC25A26	ENSG00000144741.17	0.313361	4.03E-06	1.880853
chr7_128814638_128814919_+*	CCDC136	ENSG00000128596.17	0.398132	0.000935484	2.450661

**Oligodendrocytes**					
**exon**	**gene**	**ENSEMBL ID**	**ΔΨ**	**FDR**	**log-odds ratio**
chr12_101684382_101684424_+*	MYBPC1	ENSG00000196091.15	-0.358333	0.004529261	-3.237468
chr4_76078765_76079141_+*	ART3	ENSG00000156219.16	-0.256324	0.004886756	-1.558628
chr4_51911070_51911174_+*	DCUN1D4	ENSG00000109184.15	-0.245228	0.02128649	-1.574771
chr2_64581190_64581273_+*	AFTPH	ENSG00000119844.15	-0.202684	0.009169317	-1.483841
chr11_132330153_132330185_+*	NTM	ENSG00000182667.14	0.200588	0.000216275	1.207433
chr1_19342752_19342864_-*	CAPZB	ENSG00000077549.19	0.207029	0.008267295	1.413691
chr13_111280272_111280350_+*	ARHGEF7	ENSG00000102606.18	0.227921	0.003996709	1.392706
chr2_95919733_95919805_-*	ANKRD36C	ENSG00000174501.14	0.277164	2.76E-15	2.320285
chr3_101728836_101728937_+*	CEP97	ENSG00000182504.11	0.282171	0.0462356	2.906891
chr2_95919892_95919920_-*	ANKRD36C	ENSG00000174501.14	0.284808	5.74E-15	2.400676
chr7_128812708_128812929_+*	CCDC136	ENSG00000128596.17	0.32183	0.03473503	2.067048
chr7_128814638_128814919_+*	CCDC136	ENSG00000128596.17	0.338051	0.01411319	2.063353
chrX_134485467_134485507_+*	HPRT1	ENSG00000165704.15	0.339286	0.02984758	2.058894
chr7_128815614_128815931_+*	CCDC136	ENSG00000128596.17	0.369792	0.001375935	2.243926
chr10_121899866_121899994_-	ATE1	ENSG00000107669.17	0.375947	0.01182179	4.452512

**Microglia**					
**exon**	**gene**	**ENSEMBL ID**	**ΔΨ**	**FDR**	**log-odds ratio**
chr7_94587774_94587812_-*	SGCE	ENSG00000127990.19	-0.495238	0.001933315	-3.459432
chr3_108050578_108050602_-*	CD47	ENSG00000196776.16	-0.318919	0.02618356	-2.917538
chr3_146536540_146536565_-*	PLSCR1	ENSG00000188313.13	-0.213729	5.05E-31	-2.382043

Table S3: Donor information for samples used for RT-qPCR analysis of differentially affected regions.
Clinical report of patient material used for brain-region-specific splicing analysis as measured by RT-qPCR.

	**Case 1_NBB2021-101**	**Case 2_NBB2023-142**
Age at death	76	58
Gender	Female	Female
GRN mutation	Exon 3 p.Ser82fs	Exon 2 Val8Glu
FTD-type	TDP type A	TDP type A
Cause of death	Natural death at end-stage FTD	Euthanasia
First symptoms	Daily functional impairments @ 8 years before death.	Aphasia @ ~2 years before death
Symptoms at end stage		
Motor function	Very impaired	Slightly impaired
Behavior	NA	Character change
Cognition	Very impaired (memory, concentration, orientation)	Progressive dementia
Speech	Very impaired	Very impaired
Communication	Very impaired	Slightly impaired
Tissue specifics		
Post-mortem delay	4:30 h	NA
Brain weight	735 grams	1120 grams
General remarks	Severe atrophy<br>Severe atherosclerosis<br>Ventricle dilation (including those occipitally)<br>Very small amygdala and hippocampus	Severe hypoxia due to euthanasia<br>Asymmetric brain<br>Small amygdala
Frontal cortex		
Macroscopic atrophy	Severe, mostly frontal and temporal, right more so than left.	Light to moderate, mostly frontal (none temporal), right more so than left
Tissue selected	Medial frontal gyrus 4 (within Brodmann area 6); left side	
Occipital cortex		
Macroscopic atrophy	Seemingly none	None
Tissue selected	Superior occipital gyrus 3 (Brodmann area 17); left side	
